# Impact of Metabolic Syndrome Traits on Kidney Disease Risk in Individuals with MASLD: A UK Biobank Study

**DOI:** 10.1111/liv.16159

**Published:** 2024-11-15

**Authors:** Josh Bilson, Theresa J. Hydes, Declan McDonnell, Ryan M. Buchanan, Eleonora Scorletti, Alessandro Mantovani, Giovanni Targher, Christopher D. Byrne

**Affiliations:** ^1^ School of Human Development and Health, Faculty of Medicine University of Southampton Southampton UK; ^2^ National Institute for Health and Care Research Southampton Biomedical Research Centre University of Southampton and University Hospital Southampton National Health Service Foundation Trust Southampton UK; ^3^ Department of Cardiovascular and Metabolic Medicine, 3rd Floor Clinical Sciences Centre Institute of Life Course and Medical Sciences Liverpool University Hospitals NHS Foundation Trust University of Liverpool, Longmoor Lane Liverpool UK; ^4^ University Hospital Aintree, Liverpool University Hospital NHS Foundation Trust Liverpool UK; ^5^ HPB Unit, University Hospital Southampton Southampton UK; ^6^ Primary Care and Population Sciences Faculty of Medicine University of Southampton Southampton UK; ^7^ Department of Genetics, Perelman School of Medicine University of Pennsylvania Philadelphia Pennsylvania USA; ^8^ Section of Endocrinology, Diabetes and Metabolism, Department of Medicine University and Azienda Ospedaliera Universitaria Integrata of Verona Verona Italy; ^9^ Metabolic Diseases Research Unit IRCCS Sacro Cuore—Don Calabria Hospital Negrar di Valpolicella Italy; ^10^ Department of Medicine University of Verona Faculty of Medicine and Surgery Verona Italy

**Keywords:** end‐stage renal disease, hypertension, metabolic dysfunction‐associated steatotic liver disease, type 2 diabetes

## Abstract

**Background and Aims:**

The impact of metabolic syndrome (MetS) traits on chronic kidney disease (CKD) risk in metabolic dysfunction‐associated steatotic liver disease (MASLD) is unknown. We investigated the impact of type and number of MetS traits and liver fibrosis on prevalent CKD and incident end‐stage renal disease (ESRD) risk in SLD.

**Methods:**

234 488 UK Biobank participants' were analysed. Hepatic steatosis index (> 36 for SLD, < 30 for no SLD) and MRI‐proton density fat fraction (≥ 5.56%) were used to identify SLD. MetS traits were identified using MASLD criteria. Advanced fibrosis (FIB‐4 score > 2.67) was determined using FIB‐4 scores. eGFR < 60 mL/min/1.73 m^2^ or albuminuria > 3 mg/mmol identified prevalent CKD. A validated algorithm identified incident ESRD. Binary logistic and Cox regressions were used to test associations with prevalent CKD ([adjusted odds ratios (ORs)]) and incident ESRD (adjusted hazard ratios [HRs]) respectively.

**Results:**

102 410 participants (41.2%) had SLD. 64.4% had MetS. 1.3% had FIB‐4 score > 2.67. With SLD and only one MetS trait, hypertension (OR 1.35, 95% CI 1.35–1.72) or type 2 diabetes (T2D) (OR 1.89, 95% CI 1.06–3.38) increased risk of prevalent CKD. MetS (≥ 3 traits) increased prevalent CKD risk (OR 1.94, 95% CI 1.75–2.15), which was further increased by advanced liver fibrosis (OR 4.29, 95% CI 3.36–5.47). CKD prevalence increased with increasing MetS traits. Over 13.6 years (median follow‐up), MetS was associated with increased risk of developing ESRD (HR 1.70, 95% CI 1.19–2.43).

**Conclusions:**

In MASLD, hypertension, and T2D, number of MetS traits and liver fibrosis increased risk of prevalent CKD and presence of MetS increased the risk of incident ESRD.

**Abbreviations**
ALT: Alanine transaminase
AST: Aspartate transaminase
BMI: Body mass index
CKD: Chronic kidney disease
eGFR: Estimate glomerular filtration rate
ESRD: End‐stage renal disease
FIB‐4: Fibrosis‐4
HbA1c: Haemoglobin A1c
HDL: High‐density lipoprotein
HR: Hazard ratio
HSI: Hepatic steatosis index
IQR: Interquartile range
MASLD: Metabolic dysfunction‐associated steatotic liver disease
MetS: Metabolic syndrome
MRI: Magnetic resonance imaging
OR: Odds ratio
PDFF: Proton density fat fraction
PNPLA3: Patatin‐like phospholipase domain‐containing protein 3
SLD: Steatotic liver disease
T2DM: Type 2 diabetes
UACR: Urine albumin creatine ratio
UKBB: UK Biobank
WT: Wild type

**Summary**

- People living with metabolic dysfunction‐associated steatotic liver disease (MASLD) have a higher risk of developing kidney disease, however, the impact that the severity of MASLD has on the risk of kidney disease in these people is unclear.
- Our findings show that, in people with MASLD, those with more traits of metabolic dysfunction and scaring in their liver are at the highest risk of kidney disease and are more likely to develop end‐stage kidney disease.
- Using these findings, clinicians may be able to identify those people with MASLD who have the highest risk of kidney disease and implement kidney disease monitoring or treatments earlier on.

## 1 Introduction

Metabolic dysfunction‐associated steatotic liver disease (MASLD), as defined and characterised in 2023 [1], is a new term to define and characterise the metabolic dysfunction that is known to be a key feature of nonalcoholic fatty liver disease (NAFLD). MASLD is the leading cause of chronic liver disease and affects up to nearly 30% of the population worldwide [2, 3, 4]. The economic burden of MASLD is substantial and is thought to cost the UK and USA governments £5.24 billion and $103 billion, respectively [5]. A diagnosis of MASLD requires the presence of hepatic steatosis and at least one of the five metabolic syndrome (MetS) traits [1, 6]. Currently, a single MASLD risk model exists and patients with MASLD, regardless of the number and type of MetS traits present, are managed the same way in a clinical setting despite substantial disease heterogeneity. To prompt the re‐evaluation of the single MASLD risk model, evidence demonstrating the impact of MASLD severity on the risk of hepatic and extra‐hepatic diseases is urgently required.

Patients with MASLD have a significantly greater risk of developing chronic kidney disease (CKD), which is thought to affect between ~20% and 55% of patients with MASLD [7, 8]. CKD also increases the risk of cardiovascular disease, which is the leading cause of death in people with both MASLD and CKD, making CKD an important and prevalent extra‐hepatic complication of MASLD [7, 8]. Increasing numbers of MetS traits adversely affect the risk of incident CVD, mortality [9], and major adverse liver‐related outcomes [10]. However, the impact of MASLD severity, determined by the degree of metabolic dysfunction on the risk of CKD and incident end‐stage renal disease (ESRD) is currently unknown, and exploration of this is of clinical relevance because it may facilitate the identification of high‐risk MASLD patient subgroups for targeted CKD screening and intervention strategies.

Therefore, we aimed to determine whether and to what extent the presence of specific and accumulating MetS traits and advanced liver fibrosis (as non‐invasively assessed by FIB‐4 index) affected the risk of prevalent CKD and incident ESRD in people with and without SLD.

## 2 Methods

### 2.1 Study Population

The UK Biobank (UKBB) is a national prospective cohort study aimed at improving disease prevention (http://www.ukbiobank.ac.uk/about‐biobank‐uk). Over 500 000 individuals aged 40–69 years agreed to participate and were recruited between 2006 and 2010; details of participant assessment and follow‐up can be found in Data S1. At the time of analysis, mortality and hospital admission data were available to January 2023. Ethical approval for the UKBB study was granted by the Northwest Multi‐Centre Research Ethics Committee (06/MRE08/65).

### 2.2 Inclusion and Exclusion Criteria

**FIGURE 1:**
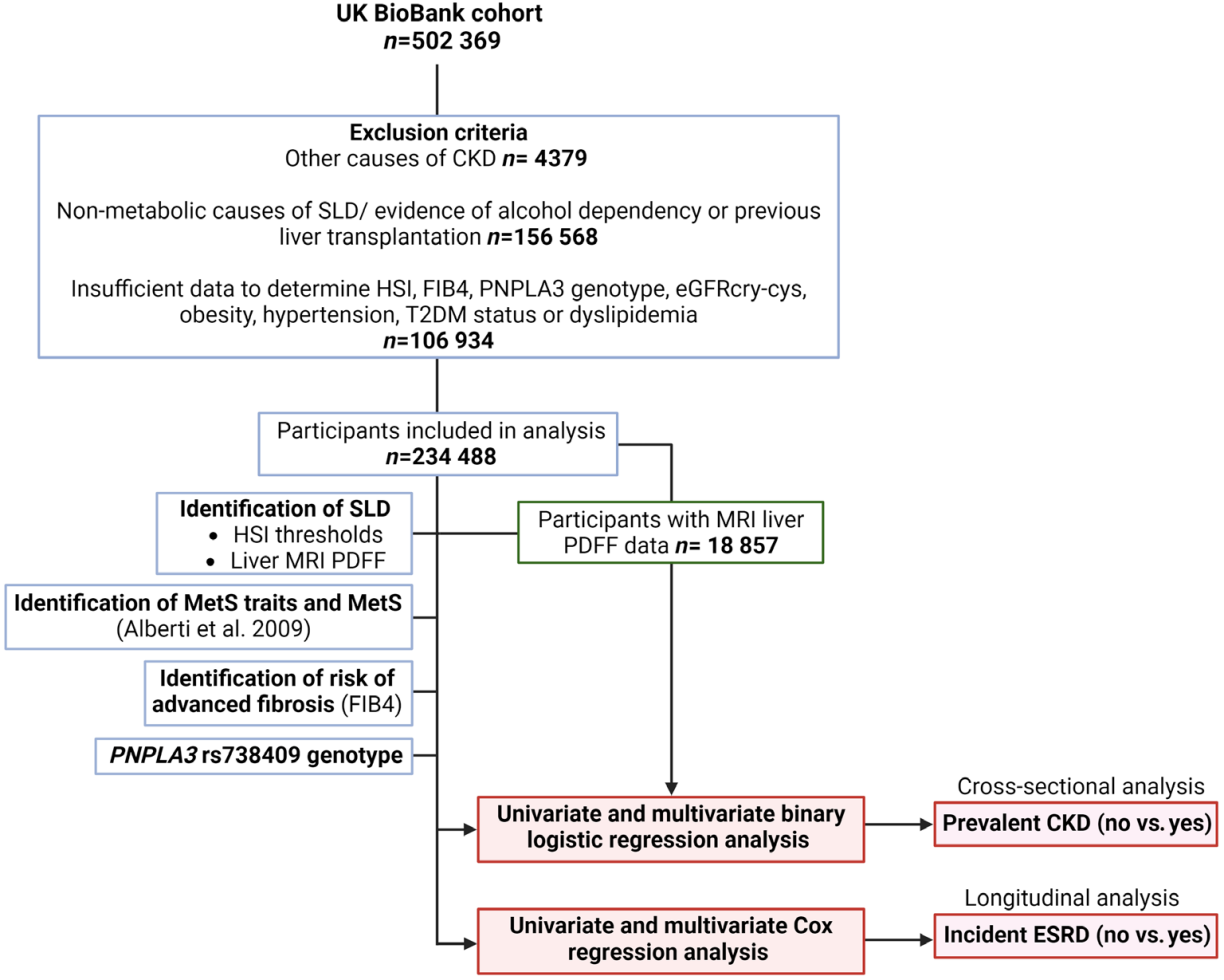
Flow chart of the study.

All UKBB participants were initially included. Details of our study design can be found in Data S1, and a study flow chart is shown in Figure 1. Participants with evidence of non‐SLD causes of liver disease, recipients of liver transplants, evidence of excess alcohol consumption/alcohol dependency or intrinsic causes of kidney disease were excluded (Methods S1). Participants for whom there were insufficient data to identify MetS traits or hepatic steatosis index (HSI) and Fibrosis‐4 (FIB‐4) were also excluded.

### 2.3 Ascertainment of Exposure

#### 2.3.1 Liver Steatosis

SLD was determined using either ICD9/ICD10 codes (Methods S1) or an HSI of > 36. Using these thresholds, the HSI has been shown to rule out SLD with a sensitivity of 93.1% and detect SLD with a specificity of 92.4% [11]. In a subgroup (*n* = 18 857), SLD was also identified using magnetic resonance imaging (MRI) proton density fat fraction (PDFF) data (≥ 5.56% for SLD).

#### 2.3.2 Metabolic Traits

Metabolic syndrome (MetS) traits were defined according to criteria published in 2009 by Alberti and colleagues [6], and the recent multi‐society Delphi consensus statement on MASLD nomenclature (Methods S1) [1]. Specific details of how MetS traits were determined can be found in Methods S1.

#### 2.3.3 Liver Fibrosis

Significant fibrosis was also defined as Fibrosis‐4 index (FIB‐4) > 2.67, and a low risk of significant fibrosis was defined as FIB‐4 < 1.3 (< 2.0 if ≥ 65 years) [12]. Participants not falling into these two groups were placed in an ‘intermediate’ fibrosis group.

#### 2.3.4 PNPLA3 Genotype

The participant's genotype for rs738409 (*PNPLA3*) (chromosome 22, location 44 324 727) was obtained using genotype data generated from DNA extracted from blood samples on either the UKBB Axiom array or the UK BiLEVE Axiom array.

### 2.4 Primary Study Outcomes

CKD was identified using a single (repeat measurements were not available) estimated glomerular filtration rate (eGFR) value of < 60 mL/min/1.73 m^2^ or a random urine albumin‐to‐creatinine ratio (UACR) ≥ 3 mg/mmol [13]. eGFR was calculated using single serum creatinine and cystatin C measurements, omitting race as per the most recent guidance [14]. We present the combined equation as this is a more valid measure [15]. Incident ESRD was defined using the UKBB ESRD algorithm. This was devised to identify participants who have had or are undergoing renal replacement therapy using ICD‐10 and OPCS‐4 codes [16].

### 2.5 Statistical Analysis

Dichotomous baseline characteristics are presented as absolute numbers (%), and continuous data are presented as median and interquartile range (IQR). Between group differences were identified using Independent‐Samples Mann–Whitney *U* tests and Fisher's Exact tests for continuous and grouped data respectively.

#### 2.5.1 Cross‐Sectional Analysis

Binary logistic regression models (odds ratios [ORs] and 95% confidence intervals [95% CI]) were used to explore the associations between the presence of SLD and the severity of MASLD with the risk of CKD at baseline. Differences in the prevalence of CKD in those with or without SLD were explored using the Fisher's Exact test. When exploring the impact of MetS traits on the risk of CKD at baseline, participants without SLD (HSI < 30) and without any MetS traits were used as a reference group. Relative to this group, we explored the change in CKD risk in participants with SLD but without any MetS traits, and then in those with SLD and any one MetS trait and continued this approach until we finally explored the risk of CKD in participants with SLD and all five MetS traits. This approach was also used in our sensitivity analysis where liver MRI PDFF measurements were used to determine the presence of SLD (≥ 5.56%). When exploring the impact of significant liver fibrosis risk, MetS and *PNPLA3* genotype on the risk of CKD, participants without SLD, without MetS, with a low risk of significant liver fibrosis, and homozygous for the wild‐type *PNPLA3* allele were used as the reference group. We then explored the impact of these exposures on the risk of CKD in patients with SLD using binary logistic regression models adjusting age, sex, ethnicity, and smoking status.

#### 2.5.2 Longitudinal Analysis

Tests of equality of ESRD event distribution between groups were undertaken using the Mantel‐cox Log Rank test. Univariable and multivariable Cox proportional hazards models were used to calculate the associations of MetS and risk of advanced liver fibrosis with incident ESRD in participants with SLD to determine hazard ratios (HR) and 95%CIs. Non‐cases were censored at the date of loss to follow‐up, date of death or the end of follow‐up (the last ICD10 update of the dataset used within this analysis was 31/10/2022). Unadjusted and adjusted HRs (adjusting for age, sex, smoking status, ethnicity, *PNPLA3* genotype and, significant liver fibrosis risk) of the association between MetS and risk of incident ESRD were calculated in participants with SLD. The validity of the proportional hazards assumption for each variable was determined by the proportional hazards assumption test based on Schoenfeld residuals (a test result of *p* > 0.05 was used to confirm variable validity). Data analyses were performed in Statistical Package for the Social Sciences (SPSS) Version 26.0 (New York, USA) and STATA Version 18.0 (Texas, USA). The Strengthening the Reporting of Observational Studies in Epidemiology (STROBE) guidelines were followed in reporting the results of this study [17].

## 3 Results

### 3.1 Baseline Demographics

A total of 234 488 UKBB participants were included in the study (Figure 1). 34 950 participants did not have SLD (HSI < 30), 102 420 (43.7%) participants had SLD (HSI > 36 or by code) and a further 97 118 participants had an indeterminate HSI. The characteristics of participants stratified by the presence or absence of SLD are shown in Table 1.

**TABLE 1:** Characteristics of participants stratified by the presence or absence of SLD.

Participant characteristics	Without SLD (HSI < 30)	With SLD (HSI > 36 or with code)	*p*
Number of participants, *n* (%)	34 950	102 420	NA
Age at attendance (years)	55.0 (14.0)	58.0 (12.0)	< 0.0001
Men, *n* (%)	12 636 (36.2)	38 987 (38.1)	< 0.0001
Smoking status			
Prefer not to answer, yes (%)	93 (0.3)	492 (0.5)	< 0.001
Never, yes (%)	23 108 (66.2)	58 610 (57.3)	< 0.0001
Previous, yes (%)	8.622 (24.7)	34 407 (33.6)	< 0.0001
Current, yes (%)	3088 (8.8)	8750 (8.6)	< 0.001
BMI (kg/m^2^)	21.8 (2.3)	30.5 (5.0)	< 0.0001
Waist circumference (cm)	74.0 (13.0)	98.0 (16.0)	< 0.0001
Systolic BP (mmHg)	129.0 (25.0)	141.0 (25.0)	< 0.0001
Diastolic BP (mmHg)	76.0 (13.0)	84.0 (14.0)	< 0.0001
HbA1c (mmol/L)	34.2 (4.3)	36.6 (6.2)	< 0.0001
TAG (mmol/L)	1.1 (0.6)	1.8 (1.2)	< 0.0001
HDL‐C (mmol/L)	1.6 (0.5)	1.3 (0.4)	< 0.0001
Number of MetS traits, *n*	1.0 (1.0)	3.0 (2.0)	< 0.0001
BMI trait, *n* (%)	985 (2.8)	100 634 (98.3)	< 0.0001
Waist circumference trait, *n* (%)	1839 (5.3)	92 831 (90.6)	< 0.0001
Hypertension trait, *n* (%)	19 687 (56.3)	83 054 (81.1)	< 0.0001
High TAG trait, *n* (%)	5228 (15.0)	55 734 (54.4)	< 0.0001
Low HDL‐C trait, *n* (%)	2989 (8.6)	36 550 (35.7)	< 0.0001
Dysglycaemia/T2D trait, *n* (%)	1428 (4.1)	33 734 (32.9)	< 0.0001
MetS, yes (%)	1212 (3.5)	65 961 (64.4)	< 0.0001
HSI	28.5 (2.1)	40.3 (6.1)	< 0.0001
FIB‐4 high risk of advanced liver fibrosis, *n* (%)	1348 (3.9)	1362 (1.3)	< 0.0001
*PNPLA3‐I148M* genotype, CC/GC/GG	21 187/12 060/1703	62 696/34 645/5079	0.07

*Note:* Data are shown as median (IQR).

Abbreviations: BMI, body mass index; BP, blood‐pressure; FIB‐4, Fibrosis‐4; HbA1c, haemoglobin A1c; HDL‐C, high‐density lipoprotein cholesterol; HSI, hepatic steatosis index; MetS, metabolic syndrome; PNPLA3, Patatin‐like phospholipase domain‐containing protein 3;T2D, type 2 diabetes; TAG, triglyceride.

### 3.2 Increased Numbers of MetS Traits and Significant Liver Fibrosis Are Associated with a Higher Prevalence of CKD

**FIGURE 2:**
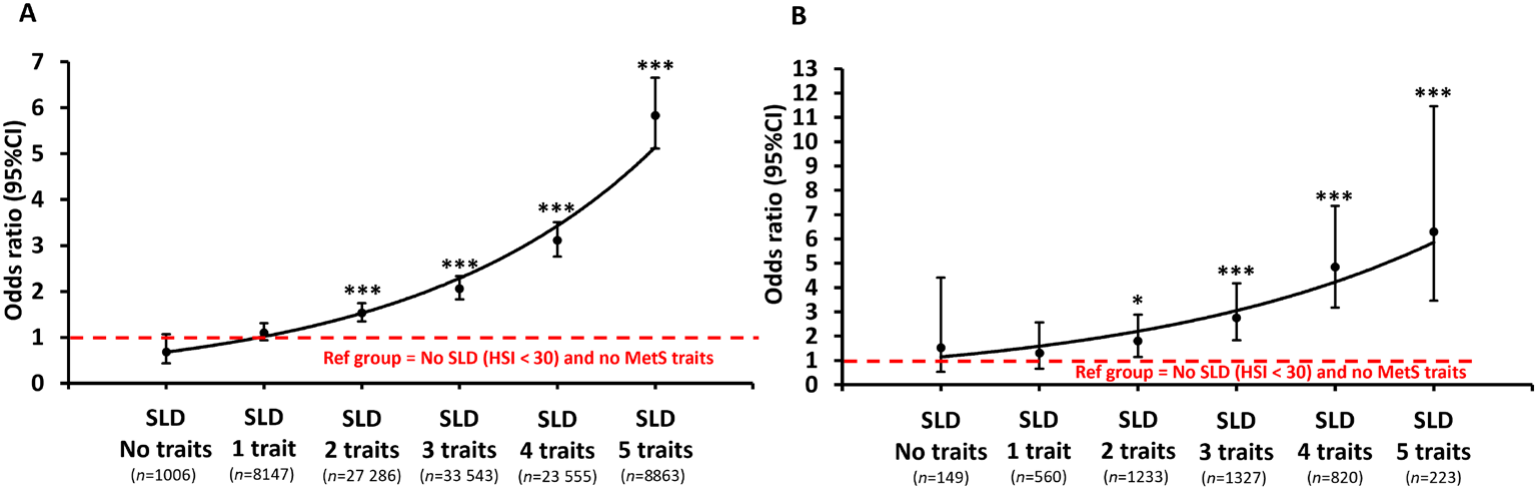
Associations between MASLD phenotype and risk of prevalent CKD. (A) Scatterplot showing the adjusted ORs (95% CIs) of the associations between increasing MetS traits and the risk of prevalent CKD in participants with SLD (HSI > 36 or code). Reference group were those without SLD (HSI < 30) or any MetS traits (*n* = 11 521). (B) Scatterplot showing the adjusted ORs (95% CIs) of the associations between increasing MetS traits and the risk of CKD in participants with SLD (MRI PDFF ≥ 5.56%). Reference group were those without SLD (MRI PDFF < 5.55%) or any MetS traits (*n* = 2698). Statistical significance denotes a significant association between the number of MetS traits and risk of prevalent CKD, which was independent of age, sex, smoking status, ethnicity, liver fibrosis (FIB‐4 thresholds) and *PNPLA3* I148M genotype. Trendlines are exponential. *p < 0.05, **p < 0.001, ***p < 0.0001. HSI, hepatic steatosis index; and MetS, metabolic syndrome; SLD, steatotic liver disease.

The presence of CKD was greater in participants with SLD (HSI > 36 or code) compared to those without SLD (HSI < 30) (Table S1). The association between SLD and prevalent CKD was also observed in a subgroup of individuals using an MRI liver PDFF threshold of ≥ 5.56% for the identification of SLD (Table S1). Given this, we explored the impact of increasing numbers of MetS traits on the risk of prevalent CKD in individuals with SLD. The characteristics of participants with SLD (HSI > 36) alone, SLD and any one MetS trait (N.B defining MASLD) and SLD with two or more MetS traits are shown in Table S2. Participants with SLD and any two MetS traits were at greater risk of prevalent CKD (OR 1.53, 95% CI 1.35–1.74, *p* < 0.0001) when compared to participants without SLD and no MetS traits independently of age, sex, smoking status, ethnicity, risk of advanced liver fibrosis determined by FIB‐4 and *PNPLA3* rs738409 genotype (Table 2). Moreover, the risk of prevalent CKD increased in parallel to the number of MetS traits present amongst participants with SLD (Table 2 and Figure 2a). In the fully adjusted analysis, we found that participants with SLD and all five MetS traits were at the highest risk of having CKD (OR 5.83, 95% CI 5.11–6.55, *p* < 0.0001) compared to those without SLD and no MetS traits (Table 2 and Figure 2a). Importantly, these findings were reproduced when we repeated this analyses in a subgroup of participants where the presence of SLD was defined using liver MRI PDFF (≥ 5.56%) (Table 3 and Figure 2b). We also found that the risk of prevalent CKD was higher in participants with SLD (HSI > 36) and any two MetS traits compared to participants with SLD any one MetS trait (OR 1.40, 95% CI 1.22–1.60, *p* < 0.0001) (Table S3). When considering specific single MetS traits, in adjusted analysis, only the presence of dysglycemia/T2D (OR 1.89, 95%C I 1.06–3.38, *p* < 0.05) and hypertension (OR 1.35, 95% CI 1.05–1.72, *p* < 0.05) were significantly associated with risk of CKD in those with SLD (HSI > 36) and only one MetS trait (i.e., minimum requirement for a MASLD diagnosis) compared to those without SLD (HSI < 30) and no MetS traits (Table 2). Moreover, we found that in participants with SLD with two, three or four MetS traits, the combination of dysglycaemia/T2DM + hypertension had a greater relative contribution to the overall burden of MetS traits in people who had compared to those who didn't have CKD (Table S4).

**TABLE 2:** Associations between MASLD phenotype and prevalent CKD.

MASLD phenotype	Participants	Case of CKD (rate per 1000 person)	Univariate OR (95% CI)	Adjusted OR (95% CI)
HSI < 30 + no components	11 521	359 (31.2)	1.00	1.00
HSI > 36 + no components MetS	1006	20 (19.9)	0.63 (0.40–0.99)*	0.68 (0.43–1.07)
HSI > 36 + any one component MetS	8167	279 (32.9)	1.10 (0.94–1.29)	1.10 (0.94–1.31)
HSI > 36 + any 2 components MetS	27 286	1465 (53.7)	1.76 (1.57–1.98)***	1.53 (1.35–1.74)***
HSI > 36 + any 3 components MetS	33 543	2567 (76.5)	2.58 (2.30–2.88)***	2.06 (1.83–2.33)***
HSI > 36 + any 4 components MetS	23 555	2654 (112.7)	3.95 (3.53–4.42)***	3.11 (2.76–3.51)***
HSI > 36 + any 5 components MetS	8863	1602 (180.8)	6.86 (6.10–7.72) ***	5.83 (5.11–6.65)***
HSI > 36 + central obesity only	4802	157 (32.7)	1.02 (0.84–1.23)	0.98 (0.81–1.2)
HSI > 36 + hyperglycaemia/T2DM only	234	13 (55.6)	1.72 (0.98–3.04)	1.89 (1.06–3.38)*
HSI > 36 + hypertension only	2248	93 (41.4)	1.29 (1.02–1.62)*	1.35 (1.05–1.72)*
HSI > 36 + high TG only	428	10 (23.4)	0.73 (0.39–1.37)	0.97 (0.51–1.85)
HSI > 36+ low HDL‐C only	176	6 (34.1)	1.06 (0.47–2.40)	1.17 (0.51–2.67)

*Note:* Fully adjusted model was adjusted for age, sex (male vs. female), smoking status (Prefer not to answer, Never, Previous, Current), ethnicity, risk of advanced liver fibrosis (FIB‐4; low, intermediate, high) and *PNPLA3* genotype (CC, GC, GG).

Abbreviations: CI, confidence intervals; CKD, chronic kidney disease; HSI, hepatic steatosis index and MASLD, metabolic dysfunction‐associated steatotic liver disease.

* *p* < 0.05, ** *p* < 0.001, *** *p* < 0.0001.

**TABLE 3:** Association between MASLD phenotype and risk of prevalent CKD in participants with MRI liver PDFF data.

MASLD phenotype	Participants	Case of CKD (rate per 1000 person)	Univariate OR (95% CI)	Adjusted model or (95% CI)
MRI PDFF < 5.55% + no components	2698	51 (18.9)	1.00	1.00
MRI PDFF ≥ 5.56% + no components MetS	149	4 (26.9)	1.43 (0.51–4.02)	1.53 (0.53–4.41)
MRI PDFF ≥ 5.56% + any one component MetS	560	12 (21.4)	1.14 (0.60–2.15)	1.31 (0.67–2.56)
MRI PDFF ≥ 5.56% + any 2 components MetS	1233	38 (30.8)	1.66 (1.09–2.55)*	1.81 (1.14–2.88)*
MRI PDFF ≥ 5.56% + any 3 components MetS	1327	66 (49.7)	2.72 (1.87–3.94)***	2.76 (1.83–4.17)***
MRI PDFF ≥ 5.56% + any 4 components MetS	820	63 (76.8)	4.32 (3.00–6.30)***	4.84 (3.18–7.36)***
MRI PDFF ≥ 5.56% + any 5 components MetS	223	21 (94.2)	5.40 (3.18–9.15)***	6.29 (3.46–11.46)***

*Note:* Fully adjusted model was adjusted for age, sex (male vs. female), smoking status (Prefer not to answer, Never, Previous, Current), ethnicity, risk of advanced liver fibrosis (FIB‐4; low, intermediate, high) and PNPLA3‐148 genotype (CC, GC, GG).

* *p* < 0.05, ** *p* < 0.001, *** *p* < 0.0001.

The characteristics of participants with SLD stratified by the presence or absence of MetS (i.e., ≥ 3 MetS traits) are reported in Table S5. In the fully adjusted analysis, the presence of MetS in participants with SLD was associated with an increased risk of prevalent CKD where SLD was determined using a HSI of > 36 or using a liver MRI PDFF threshold of ≥ 5.56% (Table 4). Next, we explored the potential impact of the risk of advanced liver fibrosis and carriage of *PNPLA3‐I148M* on the risk of prevalent CKD in participants with SLD (HSI > 36). Whilst carriage of the PNPAL3‐I148M variant was not associated with an increased risk of prevalent CKD, in the fully adjusted analysis, participants with both MetS and significant liver fibrosis (FIB‐4 > 2.67) had the highest risk of prevalent CKD compared to those without any of the explored risk factors (SLD, MetS, risk of liver fibrosis or *PNPLA3‐I148M*) (OR 4.29, 95% CI 3.36–5.47, *p* < 0.0001) regression analyses (Table 5). The interaction between MetS and advanced liver fibrosis in participants with SLD was not associated with the risk of prevalent CKD (OR 0.81, 95% CI 0.57–1.15, *p* = 0.24).

**TABLE 4:** Associations between MetS and prevalent CKD in participants with SLD.

	Number of participants	Cases of CKD (rate per 1000 person)	Univariate OR (95% CI)	Adjusted model
SLD (HSI > 36 or code)				
No MetS	36 459	1764 (48.4)	1.00	1.00
With MetS	65 961	6823 (103.4)	2.27 (2.15–2.40)***	1.91 (1.57–2.33)***
Liver MRI PDFF (≥ 5.56%)				
No MetS	1932	54 (28.0)	1.00	1.00
With MetS	2220	150 (68.2)	2.35 (1.71–3.23)***	2.36 (1.71–3.26)***

*Note:* Fully adjusted model was adjusted for age, sex (male vs. female), smoking status (Prefer not to answer, Never, Previous, Current), ethnicity, risk of advanced liver fibrosis (FIB‐4; low, intermediate, high) and *PNPLA3‐148* genotype (CC, GC, GG). Outcome variable was absence vs. presence of CKD (0 vs. 1 respectively).

*p < 0.05, **p < 0.001, ***p < 0.0001.

**TABLE 5:** Associations between SLD, MetS, risk of advanced liver fibrosis, and *PNPLA3‐*I148M with prevalent CKD.

	Number of participants	Cases of CKD (rate per 1000 person)	Univariate OR (95% CI)	Adjusted OR (95% CI)
No SLD No MetS Low risk of significant fibrosis *PNPLA3* (C/C)	10 285	451 (43.9)	1.00	1.00
SLD only	16 874	770 (45.6)	1.04 (0.93–1.17)	0.97 (0.86–1.09)
SLD + MetS	30 916	3076 (99.5)	2.41 (2.18–2.67)***	1.94 (1.75–2.15)***
SLD + *PNPLA3* (G/G)	1359	59 (43.4)	0.99 (0.75–1.31)	0.93 (0.70–1.23)
SLD + high risk of significant fibrosis	221	26 (117.6)	2.91 (1.91–4.43)***	2.12 (1.38–3.27)***
SLD + MetS + high risk of significant fibrosis	537	118 (219.7)	6.14 (4.90–7.69)***	4.29 (3.36–5.47)***

*Note:* Fully adjusted model was adjusted for age, sex (male vs. female), smoking status (Prefer not to answer, Never, Previous, Current), and ethnicity. Outcome variable was absence vs. presence of CKD (0 vs. 1 respectively).

Abbreviations: CI, confidence intervals; CKD, chronic kidney disease; HSI, hepatic steatosis index; MetS, metabolic syndrome; PNPLA3, Patatin‐like phospholipase domain‐containing protein 3 and SLD, steatotic liver disease.

*p < 0.05, **p < 0.001, ***p < 0.0001.

### 3.3 Increased MASLD Severity Is Associated with a Greater Risk of Incident ESRD

We next explored the impact of MetS and risk of advanced liver fibrosis on the risk of incident ESRD in participants with SLD. A total of 102 420 participants were followed for a median (IQR) of 13.6 (1.5) years, and there was a total of 229 cases of incident ESRD during this time median follow‐up time in those who developed incident ESRD was 7.6 (5.4) years. It is important to note that in the subgroup of participants with liver MRI PDFF data, only 10 participants developed incident ESRD, consequently. Nevertheless, the ESRD event distribution in participants with SLD (HSI > 36) without vs. with MetS was significantly different *χ* ^2^(1) = 39.14, *p* < 0.0001. In the fully adjusted Cox regression model, MetS was independently associated with a higher risk of developing incident ESRD (HR 1.70, 95% CI 1.19–2.43, *p* = 0.004) in participants with SLD (Table 6). It is also important to note that only eight participants with SLD and advanced liver fibrosis (FIB‐4 > 2.67) developed incident ESRD during the follow‐up.

**TABLE 6:** Association between MetS and incident ESRD in people with SLD.

	Participants	Cases of incident ESRD (rate per 1000 person)	Univariate HR (95% CI)	Adjusted HR Model 2	Adjusted HR Model 3
SLD no MetS	36 459	37 (1.02)	1.00	1.00	1.00
SLD with MetS	65 961	192 (2.91)	2.92 (2.05–4.15)***	2.40 (1.68–3.43)***	1.70 (1.19–2.43)*

*Note:* Model 2 was adjusted for age, sex (male vs. female), smoking status (Prefer not to answer, never, previous, current), ethnicity, risk of advanced liver fibrosis (FIB‐4; low, intermediate, high) and *PNPLA3*‐I148M genotype (CC, GC, GG). Model 3 was adjusted for the same factors included in model 2 *plus* presence of CKD at baseline. The outcome variable was absence vs. presence of incident ESRD (0 vs. 1 respectively).

Abbreviations: ESRD, end‐stage renal disease; HIS, hepatic steatosis index; HR, hazard ratio; MetS, metabolic syndrome; SLD, steatotic liver disease.

*p < 0.05, **p < 0.001, ***p < 0.0001.

## 4 Discussion

Using the large UKBB database, we show for the first time that the risk of prevalent CKD in participants with SLD increases with increasing numbers of MetS traits. In participants with SLD and only one MetS trait (the minimum requirement to assign a MASLD diagnosis), only the presence of hypertension or dysglycaemia/T2DM was independently associated with an increased risk of prevalent CKD. Moreover, we show that the risk of CKD is markedly increased with the presence of all five MetS traits and is the highest in participants with a combination of SLD, MetS and significant liver fibrosis (FIB‐4 > 2.67). Finally, we also show that the presence of MetS in participants with SLD is strongly and independently associated with an increased risk of developing incident ESRD during a median follow‐up of 13.6 years.

Our findings show the significant and marked impact of MetS trait numbers on CKD risk and highlight an important limitation of the single MetS trait ‘MASLD model’ to adequately capture the heterogeneity of the different subgroups of MASLD on the risk of CKD and ESRD. We validated our data by showing the positive association between the number of MetS traits/the presence of MetS and the risk of prevalent CKD in participants with SLD using the highly sensitive MRI liver PDFF measurements. Our findings also support the notion that people with SLD and two MetS traits are at a higher risk of MASLD complications compared to those with SLD and only one MetS trait N.B the minimum required for a MASLD diagnosis to be made supporting recent work from others [18]. Importantly, we showed that the risk of prevalent CKD is the highest in participants with a combination of SLD, MetS, and significant liver fibrosis when compared to participants without any of these risk factors, and even after all adjustments, there was an approximate 4.3‐fold increase in the risk of CKD. Moreover, using a longitudinal study design, we found that the presence of MetS in people with SLD also increased the risk of incident ESRD even after adjusting for any pre‐existing evidence of CKD. These findings highlight an important high‐risk subgroup of patients with MASLD (i.e., those patients with MASLD who have multiple MetS traits and a high probability of advanced liver fibrosis) where targeted CKD screening and interventions could be implemented with a potential high cost‐effectiveness.

Our analyses indicate that of the five MetS traits, only hypertension and dysglycaemia/T2D significantly increased the risk of prevalent CKD in participants with SLD who had only one MetS trait (i.e., the minimum requirement to assign a MASLD diagnosis) [1]. This was further supported by our observation that the combination of dysglycaemia/T2DM and hypertension contributed a larger amount towards the total MetS trait burden in people with SLD and CKD compared to those with SLD but without CKD. It is well‐established that hypertension and T2D are important risk factors for the development of CKD and ESRD [8, 19]. Indeed, meta‐analysis of 11 studies carried out in 2011 indicated that hypertension and impaired fasting glucose were both associated with an increased risk of CKD [20]. Similarly, hypertension was associated with an accelerated decline in renal function over a 3‐year follow‐up in a general population cohort [21]. Whilst clinical trials have generally found that intensive blood‐pressure lowering does not result in a lower rate of renal function decline [22, 23], blood‐pressure‐lowering interventions reduce the risk of adverse cardiovascular outcomes and mortality in patients with CKD [24]. Emerging evidence also indicates the potential efficacy of anti‐hyperglycemic treatments in lowering the risk of adverse cardiovascular and renal outcomes in patients with T2DM and CKD [25, 26, 27]. Noteworthy, semaglutide may be an effective treatment strategy for the management of MASLD which [28]. In 2024, semaglutide was also shown to reduce the risk of clinically important renal outcomes and death from cardiovascular causes in patients with T2D and CKD [25]. Notably, we included the prescription of anti‐hypertensive and anti‐hyperglycemic treatments as evidence of hypertension and T2DM respectively to help define MetS traits as key exposures of interest in our study. Consequently, we did not adjust for these drug treatments in the regression models. However, taking considering our findings in context with the evidence above, the implementation of anti‐hypertensive or anti‐hyperglycemic therapies in individuals with SLD and hypertension or T2D may lead to a reduction in the risk of CKD and adverse cardiovascular outcomes.

Previous work from others have described the potentially important role of visceral obesity, low HDL‐C, and high TAG concentrations as factors contributing to renal damage and CKD risk [29, 30]. The presence of low HDL‐C and high TAG MetS traits is reflective of a pro‐atherogenic lipoprotein phenotype which is known to cause cardiovascular disease, exacerbate systemic inflammation, and promote the formation of atherogenic plaques [31]. Given that CVD is a strong CKD risk factor, the detrimental effects of lipid associated MetS traits may contribute to renal damage and exacerbate CKD risk in people with SLD. Similarly, visceral obesity drives systemic inflammation which, along with derangements in adipokine profiles and lipid handling, may induce haemodynamic changes, renal dysfunction, and promote the development and progression of CKD. That said, it is important to acknowledge that it remains unclear whether the pro‐atherogenic or visceral obesity MetS traits are causally linked to CKD or are reflective of the decline in overall metabolic health typically observed in individuals with CKD. Indeed, whilst we did not identify any associations between low HDL‐C, high TAG or visceral obesity and the risk of prevalent CKD within our study, it is important to highlight that the event rates in these analyses were relatively small and should not be interpreted as unequivocal evidence that these traits do not impact CKD risk in individuals with SLD.

With establishing the diagnostic criteria for MASLD in 2023, the findings of our study highlight the need to re‐evaluate the current single MASLD risk model and move towards a clinical approach that considers both the number and type of MetS traits present in the affected. Such re‐evaluation could lead to a risk stratified MASLD model where high‐risk subgroups of patients with MASLD are better identified, enabling the appropriate monitoring, assessment, and treatment to decrease the risk of CKD and CKD‐associated cardiovascular complications. Critically, the findings of our study may also translate to other extra‐hepatic complications of MASLD. Thus, the utility of a risk stratified MASLD model could enable clinicians to identify high‐risk MASLD subgroups and implement appropriate treatment strategies for diseases beyond CKD.

The current study has strengths with assessment of MetS traits and MetS as exposures of interest and both CKD and ESRD as key outcomes in both cross‐sectional and retrospective cohort study analyses. However, some important limitations should be considered. Although, we used previously validated HSI thresholds to identify SLD, it is worth highlighting, however, that a previous study has shown that at HSI < 30 or > 36 values, these thresholds have a sensitivity of 93.1% and a specificity of 92.4%, respectively, for diagnosing SLD That said, we also performed a sensitivity analysis using highly sensitive liver MRI PDFF data to determine the presence of SLD, and our findings were comparable. Secondly, we determined the presence of CKD using a single measurement of eGFR or albuminuria and could not verify that the selected participants had abnormal biochemistry for at least 3 months. This may have resulted in a small number of false‐positive diagnoses of CKD within our study, but any misclassification bias would have attenuated the strength of our findings towards the null. There were also a relatively small number of participants within our study with SLD and a raised FIB‐4 score who also developed incident ESRD (*n* = 8). Consequently, our study likely lacked sufficient power to detect an association between liver fibrosis severity and the risk of incident ESRD. Another potential limitation of our study is that follow‐up eGFR measurements were not available for participants and we relied on the algorithmically defined outcome definition of ESRD which was developed and validated by the UK Biobank Outcome Adjunction Group in conjunction with clinical experts. Whilst this algorithm has been shown to successfully identify people with ESRD in a separate UK cohort [32], it includes the use of ICD‐10 codes which typically thought to underestimate disease incidence and prevalence. Potentially due to this limitation, we were unable to explore the additive effect of increasing numbers of MetS traits on the risk of incident ESRD in participants with SLD. Whilst not currently included as a MetS trait characterising MASLD [1, 6], we were not able to explore the impact of insulin resistance on the risk of CKD since fasted glucose and insulin concentrations are not available within the UK BioBank cohort. Moreover, changes in MetS traits during the follow‐up period were not recorded and we were unable to consider the impact that these changes may have had on the risk of incident ESRD in people with SLD. Further work is also required to determine the external validity of our findings obtained from a predominantly White Northern European cohort of participants. Whilst in our study, the presence of PNPLA3‐I148M variant did not increase the risk of CKD, it should be noted that the number of participants included in this analysis was small (*n* = 1359) and of these participants there were only 59 cases of CKD. Consequently, additional work is required to elucidate the potential impact of genetic risk factors on the risk of CKD in people with MASLD and whether these modify the impact of MetS traits CKD/ESRD risk in people with MASLD.

In conclusion, using the large UKBB database, we found that an increasing number of MetS traits and a high probability of advanced liver fibrosis were independently associated with an increased risk of prevalent CKD in subjects with MASLD. We identified that hypertension and dysglycaemia/T2D were the only two MetS traits that increased the risk of prevalent CKD in people affected by SLD and only one MetS trait (the minimum requirement for a MASLD diagnosis to be made). Moreover, and importantly for clinical practice, the presence of MetS in subjects with SLD was independently associated with an increased risk of developing incident ESRD. These findings highlight the importance of characterising and defining the severity of metabolic dysfunction in each subject with MASLD rather than diagnosing a subject with MASLD, simply because they have SLD and a single MetS trait, and thereby not attempting to evaluate the other easily measured MetS traits. These findings may also translate to other extra‐hepatic manifestations of SLD and prompt the need to re‐evaluate the current single MetS trait MASLD model.

## Author Contributions

J.B., T.J.H., and C.D.B. were involved in the conception of the study. J.B. and C.D.B. were involved in the analysis and interpretation of the results and wrote the first draft of the manuscript. J.B., T.J.H., D.M., E.S., A.M., G.T., and C.D.B. were involved in the interpretation of the results and contributed to the discussion. All authors edited, reviewed, and approved the final version of the manuscript. C.B.D. is the guarantor of the study who accepts full responsibility for the work and the conduct of the study, has access to the data, and controlled the decision to publish.

## Conflicts of Interest

C.D.B. and R.M.B. have an independent research grant from Echosens. None of the other authors have any conflicts of interest to declare.

## Supporting information


Data S1.

Methos S1.

## Data Availability

The authors have nothing to report.
