# Supplementary material for: Impact of Metabolic Syndrome Traits on Kidney Disease Risk in Individuals with MASLD: A UK Biobank Study
Source: Liver Int. 2024 Nov 15;45(4):e16159. doi: 10.1111/liv.16159 (PMC11897864; doi:10.1111/liv.16159)
Supplement: Supplementary file 1 — Data S1. [file LIV-45-0-s002.docx]

| Supplementary Table 1. Prevalence of CKD was higher in those with evidence of SLD compared to those without evidence of SLD | | | | |
| --- | --- | --- | --- | --- |
|  |  | **Participants with evidence of CKD, *n* (%)** | |  |
|  | **Total number of participants** | **Without SLD** | **With SLD** | ***P* value** |
| SLD, no vs yes (HSI <30 vs >36 or code) | 137,370 | 1,645 (4.7) | 8,587 (8.4) | P<0.00001 |
| Fatty liver by liver MRI PDFF (<5.55% vs ≥5.56%) | 18,857 | 484 (3.3) | 204 (4.7) | P<0.0001 |
| Between group comparisons were made using the Fisher’s Exact test. | | | | |

| Supplementary Table 2. Participant characteristics of those with SLD, SLD with one MetS and SLD with more than one MetS traits | | | |  |
| --- | --- | --- | --- | --- |
| **Participant characteristics** | **SLD (HSI >36)** | **SLD with one MetS trait** | **SLD with more than 1 MetS traits** | **P value** |
| Number of participants, n (%) | 102,420 | 8,167 | 93,247 |  |
| Age at attendance (years) | 58.0 (12.0) | 53.0 (13.0) | 59.0 (13.0) | <0.0001 |
| Men, n (%) | 38,987 (38.1) | 2,478 (30.3) | 36,196 (38.8) | <0.0001 |
| **Smoking status** |  |  |  |  |
| Prefer not to answer, yes (%) | 492 (0.5) | 36 (0.4) | 453 (0.5) | <0.001 |
| Never, yes (%) | 58,610 (57.3) | 5,128 (62.9) | 52,826 (56.7) | <0.0001 |
| Previous, yes (%) | 34,407 (33.6) | 2,455 (30.1) | 31,678 (34.0) | <0.0001 |
| Current, yes (%) | 8,750 (8.6) | 538 (6.6) | 8,139 (8.7) | <0.0001 |
|  |  |  |  |  |
| BMI (kg/m^2^) | 30.5 (5.0) | 28.9 (3.5) | 30.7 (5.0) | <0.0001 |
| Waist circumference (cm) | 98.0 (16.0) | 90.0 (13.0) | 99.0 (15.0) | <0.0001 |
| Systolic BP (mmHg) | 141.0 (25.0) | 125.0 (15.0) | 142.0 (24.0) | <0.0001 |
| Diastolic BP (mmHg) | 84.0 (14.0) | 78.0 (10.0) | 85.0 (13.0) | <0.0001 |
| HbA1c (mmol/L) | 36.6 (6.2) | 34.4 (4.3) | 37.0 (6.2) | <0.0001 |
| TAG (mmol/L) | 1.8 (1.2) | 1.2 (0.5) | 1.9 (1.2) | <0.0001 |
| HDL-C (mmol/L) | 1.3 (0.4) | 1.5 (0.4) | 1.2 (0.4) | <0.0001 |
|  |  |  |  |  |
| Number of MetS factors, n | 1.0 (1.0) | 1.0 (0.0) | 3.0 (2.0) | <0.0001 |
| BMI factor, n (%) | 985 (2.8) | 7,742 (94.8) | 92,041 (98.7) | <0.0001 |
| Waist circumference factor, n (%) | 1,839 (5.3) | 4,959 (60.7) | 87,872 (94.2) | <0.0001 |
| Hypertension factor, n (%) | 19,687 (56.3) | 2,341 (28.7) | 80,713 (86.6) | <0.0001 |
| Hypertriglyceridemia factor, n (%) | 5,228 (15.0) | 438 (5.4) | 55,296 (59.3) | <0.0001 |
| Hypocholesterolaemia factor, n (%) | 2,989 (8.6) | 182 (2.2) | 36,368 (39.0) | <0.0001 |
| Hyperglycaemia/T2DM factor, n (%) | 1,428 (4.1) | 247 (3.0) | 33,487 (35.9) | <0.0001 |
| MetS, yes (%) | 1,212 (3.5) | 0.0(0.0) | 65,961 (65.0) | <0.0001 |
| HSI | 28.5 (2.1) | 38.1 (3.3) | 40.6 (6.2) | <0.0001 |
| FIB-4 high risk of advanced liver fibrosis, n (%) | 1,348 (3.9) | 69 (0.8) | 1,288 (1.4) | <0.0001 |
| PNPLA3-148 genotype, CC/GC/GG | 21,187/ 12,060 / 1,703 | 5,003 / 2,740 /424 | 57,069 /31,586 /4,592 | 0.52 |
| Data are shown as median (IQR)  *Abbreviations*: BMI; body mass index, BP; blood pressure, HbA1c; haemoglobin A1c, TAG; triglyceride, HDL-C; high-density lipoprotein cholesterol, MetS; metabolic syndrome, T2D; type 2 diabetes, HSI; hepatic steatosis index, FIB-4; Fibrosis-4, PNPLA3; Patatin-like phospholipase domain-containing protein 3. | | | | |

| **Supplementary Table 3. Risk of prevalent CKD in participants with ‘MASLD-2’ compared to those with ‘pre-MASLD’.** | | | | |
| --- | --- | --- | --- | --- |
|  | **Number of participants** | **Cases of CKD (rate per 1,000 person)** | **Univariate OR (95%CI)** | **Adjusted OR (95%CI)** |
| SLD + any one MetS traits (i.e. ‘pre-MASLD’) | 8,167 | 279 (34.2) | 1.00 | 1.00 |
| SLD + any two MetS traits (i.e. ‘MASLD-2’) | 27,286 | 1,465 (53.7) | 1.60 (1.41-1.83) *** | 1.40 (1.22-1.60) *** |
| Fully adjusted model was adjusted for age, sex (male vs. female), smoking status (Prefer not to answer, Never, Previous, Current) and ethnicity, PNPLA3 genotype (CC vs GC vs GG) and probability of advanced liver fibrosis according to FIB-4 (low vs intermediate vs high)  Outcome variable was absence vs presence of CKD (0 vs. 1 respectively).  *** P<0.0001  *Abbreviations*: SLD; steatotic liver disease, MetS; metabolic syndrome, PNPLA3; Patatin-like phospholipase domain-containing protein 3, CKD; chronic kidney disease, CI; confidence intervals. | | | | |

| **Supplementary table 4. Relative contribution of MetS traits in participants with SLD with or without CKD stratified by number of MetS traits present.** | | | | |
| --- | --- | --- | --- | --- |
|  | **Total** | **Without CKD** | **With CKD** | **P value** |
| **2 MetS traits** | **27,286** | **25,821** | **1,465** |  |
| Hypertension | 20,610 | 19,397 (37.5) | 1213 (41.4) | <0.0001 |
| Dysglycaemia/T2DM | 2,413 | 2,258 (4.4) | 155 (5.3) | 0.009 |
| Waist circumference | 23,951 | 22,660 (43.9) | 1,291 (44.1) | 0.35 |
| Low HDL | 2,517 | 2,423 (4.7) | 94 (3.2) | <0.0001 |
| High TAG | 5,081 | 4,904 (9.5) | 177 (6.1) | <0.0001 |
| Hypertension + T2DM | 954 | 877 (1.7) | 77 (2.7) | <0.0001 |
|  |  |  |  |  |
| **3 MetS traits** | **33,543** | **30,976** | **2,567** |  |
| Hypertension | 29,043 | 26,739 (28.8) | 2,304 (29.6) | <0.0001 |
| Dysglycaemia/T2DM | 9,826 | 8,719 (9.4) | 1,107 (14.2) | <0.0001 |
| Waist circumference | 31,862 | 29,398 (31.6) | 2,464 (31.6) | 0.01 |
| Low HDL | 9,213 | 8,622 (9.3) | 591 (7.6) | <0.0001 |
| High TAG | 20,775 | 19,450 (20.9) | 1,325 (17.0) | <0.0001 |
| Hypertension + T2DM | 7,995 | 7,113 (8.3) | 882 (12.8) | <0.0001 |
|  |  |  |  |  |
| **4 MetS traits** | **23,555** | **20,901** | **2,654** |  |
| Hypertension | 22,197 | 19,691 (23.6) | 2,506 (23.6) | 0.35 |
| Dysglycaemia/T2DM | 12,475 | 10,789 (12.9) | 1,686 (15.9) | <0.0001 |
| Waist circumference | 23,196 | 20,593 (24.6) | 2,603 (24.5) | 0.05 |
| Low HDL | 15,775 | 14,129 (16.9) | 1,646 (15.5) | <0.0001 |
| High TAG | 20,577 | 18,402 (22.0) | 2,175 (20.1) | <0.0001 |
| Hypertension + T2DM | 11,162 | 9,579 (12.9) | 1,583 (17.5) | <0.0001 |
| Data are presented as absolute number of participants affected by specific trait (relative contribution/weight (%) of the specific trait to the overall burden of MetS traits in the group).  Relative contribution/weight of specific trait was determined by dividing the prevalence of the specific trait by the total prevalence of all five traits. For the joint contribution of hypertension + dysglycaemia/T2DM the prevalence of this combination of traits was subtracted from the prevalence of hypertension and dysglycaemia (to avoid double counting of prevalence). These adjusted prevalence values were then totalled and used to divide the prevalence of dysglycaemia/T2DM + hypertension to obtain a relative contribution of the combination of these MetS traits to overall burden of MetS traits in the group.  P values indicate differences between the absolute prevalence of MetS trait between participants with SLD with vs without CKD. | | | | |

| Supplementary Table 5. Participant characteristics of those with SLD with or without MetS | | |  |
| --- | --- | --- | --- |
| **Participant characteristics** | **SLD without MetS** | **SLD with MetS** | **P value** |
| Number of participants, n (%) | 36,459 | 65,961 | N/A |
| Age at attendance (years) | 56.0 (13.0) | 59.0 (12.0) | <0.0001 |
| Men, n (%) | 12,063 (33.1) | 26,924 (40.8) | <0.0001 |
| **Smoking status** |  |  |  |
| Prefer not to answer, yes (%) | 143 (0.4) | 349 (0.5) | <0.001 |
| Never, yes (%) | 22,300 (61.2) | 36,310 (55.1) | <0.0001 |
| Previous, yes (%) | 11,547 (31.7) | 22,860 (34.7) | <0.0001 |
| Current, yes (%) | 2,426 (6.7) | 6,324 (9.6) | <0.0001 |
|  |  |  |  |
| BMI (kg/m^2^) | 29.7 (4.2) | 31.1 (5.3) | <0.0001 |
| Waist circumference (cm) | 80.0 (13.0) | 89.0 (13.0) | <0.0001 |
| Systolic BP (mmHg) | 134.0 (26.0) | 143.0 (23.0) | <0.0001 |
| Diastolic BP (mmHg) | 82.0 (13.0) | 83.0 (13.0) | <0.0001 |
| HbA1c (mmol/L) | 34.9 (4.4) | 38.2 (6.8) | <0.0001 |
| TAG (mmol/L) | 1.3 (0.6) | 2.2 (1.2) | <0.0001 |
| HDL-C (mmol/L) | 1.5 (0.4) | 1.2 (0.4) | <0.0001 |
|  |  |  |  |
| Number of MetS factors, n | 2.0 (1.0) | 3.0 (1.0) | <0.0001 |
| BMI factor, n (%) | 35,329 (96.9) | 65,961 (99.0) | <0.0001 |
| Waist circumference factor, n (%) | 28,910 (79.3) | 63,921 (96.9) | <0.0001 |
| Hypertension factor, n (%) | 22,951 (63.0) | 60,103 (91.1) | <0.0001 |
| Hypertriglyceridemia factor, n (%) | 5,519 (15.1) | 50,215 (76.1) | <0.0001 |
| Hypocholesterolaemia factor, n (%) | 2,699 (7.4) | 33,851 (51.3) | <0.0001 |
| Hyperglycaemia/T2DM factor, n (%) | 2,660 (7.3) | 31,074 (47.1) | <0.0001 |
| HSI | 38.8 (4.3) | 41.3 (6.6) | <0.0001 |
| FIB-4 high risk of advanced liver fibrosis, n (%) | 376 (1.0) | 986 (1.5) | <0.0001 |
| PNPLA3-148 genotype, CC/GC/GG | 22,171 /12,402 /1,886 | 40,525 /22,243 /3,193 | 0.04 |
| Data are shown as median (IQR)  *Abbreviations*: BMI; body mass index, BP; blood pressure, HbA1c; haemoglobin A1c, TAG; triglyceride, HDL-C; high-density lipoprotein cholesterol, MetS; metabolic syndrome, T2D; type 2 diabetes, HSI; hepatic steatosis index, FIB-4; Fibrosis-4, PNPLA3; Patatin-like phospholipase domain-containing protein 3. | | | |
