# Supplementary material for: Impact of Metabolic Syndrome Traits on Kidney Disease Risk in Individuals with MASLD: A UK Biobank Study
Source: Liver Int. 2024 Nov 15;45(4):e16159. doi: 10.1111/liv.16159 (PMC11897864; doi:10.1111/liv.16159)
Supplement: Supplementary file 2 — Methos S1. [file LIV-45-0-s001.docx]

**Supplementary Material**

**Contents**

[Supplementary Methods 2](#_Toc178942463)

[UKBB cohort assessment and follow-up*.* 2](#_Toc178942464)

[Calculation of weekly alcohol consumption. 2](#_Toc178942465)

[Ascertainment of metabolic syndrome traits. 2](#_Toc178942466)

[Study design. 3](#_Toc178942467)

[Supplementary methods tables 4](#_Toc178942468)

[Supplementary Method Table 1. Codes used to identify participants with evidence of non-SLD causes of liver disease 4](#_Toc178942469)

[Supplementary Method Table 2. Codes used to identify participants with evidence of alcohol dependency 6](#_Toc178942470)

[Supplementary Method Table 3. Codes used to identify those with evidence of intrinsic causes of renal disease 7](#_Toc178942471)

[Supplementary Method Table 4. ICD-9/10 codes used to identify participants with SLD 11](#_Toc178942472)

[Supplementary Method Table 5. Variables and criteria used to identify participants with metabolic syndrome traits 12](#_Toc178942473)

[Supplementary Method Table 6. ICD10 and self-report codes used to identify participants with T2D 13](#_Toc178942474)

[Supplementary Method Table 7. Anti-hyperglycaemia medication codes used to identify those with T2D 14](#_Toc178942475)

[Supplementary Method Table 8. Codes used to exclude participants with T1DM or gestational diabetes 16](#_Toc178942476)

[Supplementary Method Table 9. Codes used to identify participants with hypertension 17](#_Toc178942477)

[Supplementary Method Table 10. Anti-hypertension medication codes used to identify participants with hypertension 18](#_Toc178942478)

[Supplementary Method Table 11. Codes used to identify participants with dyslipidaemia 25](#_Toc178942479)

[Supplementary Method Table 12. Codes of lipid-lowering medications used to identify those with dyslipidaemia 26](#_Toc178942480)

# Supplementary Methods

## UKBB cohort assessment and follow-up*.*

During baseline assessment visits, participants completed questionnaires about their demographics, medical history and lifestyle. Self-reported doctor-diagnosed medical conditions were verified and coded during a face-to-face interview (https://biobank.ctsu.ox.ac.uk/crystal/field.cgi?id=20002). Volunteers underwent a physical examination and provided blood and urine samples. All participants gave consent to be followed up through linkage to electronic health records (death and cancer records held by the Office for National Statistics and the Registrar General’s Office; hospital records held by the Department of Health’s Hospital Episode Statistics; Scottish Morbidity Records).

## Calculation of weekly alcohol consumption.

Weekly alcohol consumption (grams/week) was calculated using the frequency of different alcohols consumed by participants. Men consuming >210 grams/week and women consuming >140 grams/week of alcohol were excluded from analyses. For those without specific alcoholic drink consumption data, those consuming alcohol daily or almost daily were also excluded (Data field: 1558). The following grams of alcohol per drink were used to calculate weekly alcohol consumption/week using ‘Average weekly’ alcoholic drink consumption data: a measure of spirit = 8 grams (Data field: 1598), a glass of fortified wine = 10 grams (Data field: 1608), other alcoholic drink = 12 grams (Data field: 5364), a pint of beer of cider = 20 grams (Data field: 1588), a glass of white wine/champagne = 14 grams (Data field: 1578) and a glass of red wine = 14 grams (Data field: 1568).

## Ascertainment of metabolic syndrome traits.

Determination of the MetS traits was done in accordance with the Alberti et al. 2009 criteria ^1^. A waist circumference of >94 cm and >80 cm for men and women was used to identify participants with central obesity. Due to concerns of collinearity with the HSI, which is strongly influenced by BMI, we did not include BMI measurements in our analysis or as a MetS trait. Baseline type 2 diabetes mellitus (T2D) status was determined using ICD9/10 and self-reported codes (Supplementary Table 6) or via the identification of participants receiving anti-hyperglycemic medications (Supplementary Table 7) after excluding those participants with evidence of type 1 diabetes or gestational diabetes (Supplementary Table 8). Additionally, a hemoglobin A1c (HbA1c) of ≥39 mmol/mol was used to identify participants with dysglycemia (prediabetes) as per MASLD diagnosis guidelines ^2^. Hypertension was ascertained using ICD9/10 and self-reported codes (Supplementary Table 9) and by identifying participants receiving anti-hypertensive treatments (Supplementary Table 10) or in those where diastolic (data field: 4080) and systolic (data field: 4079) blood pressures were ≥85 or ≥130 mmHg, respectively.

Hypertriglyceridemia was identified by plasma triglyceride concentrations ≥ 1.70 mmol/l or using ICD9/ICD10 and self-reported codes (Supplementary Table 11) or the use of lipid-lowering medications (Supplementary Table 11). A low high-density lipoprotein (HDL) cholesterol concentration was determined using plasma HDL-C concentrations (data field: 30760) (≤1.0 mmol/l (men), or ≤1.3 mmol/l (women)) or using ICD9/ICD10 and self-reported codes (Supplementary Table 11) or for participants prescribed lipid-lowering medications (Supplementary Table 12). The presence of MetS was determined by the presence of ≥3 MetS traits (central obesity, hypertension, T2DM/dysglycemia, hypertriglyceridemia, and low HDL concentrations) as per criteria published by Alberti et al ^1^.

## Study design.

In our cross-sectional analysis, the association of different determinants of MASLD severity (presence of MetS traits, significant liver fibrosis assessed via the FIB-4 score and presence of *PNPLA3* rs738409 genotype (CC vs GC vs GG)) on the risk of prevalent CKD was explored adjusting for age, sex, smoking status, and ethnicity. In our longitudinal analysis, the development of incident ESRD was the primary outcome, and a retrospective cohort study design was used (Figure 1). All exposure variables were consistent with those used in the cross-sectional analyses and we also adjusted for the presence of CKD at baseline.

# Supplementary methods tables

| Supplementary Method Table 1. Codes used to identify participants with evidence of non-SLD causes of liver disease | | |
| --- | --- | --- |
| **UKBB code** | **Code type** | **Code** |
| 1604 | UKBB SR | 1604 Alcoholic liver disease / alcoholic cirrhosis |
| 1156 | UKBB SR | 1156 Infective/viral hepatitis |
| 1579 | UKBB SR | 1579 Hepatitis B |
| 1580 | UKBB SR | 1580 Hepatitis C |
| 1581 | UKBB SR | 1581 Hepatitis D |
| 1506 | UKBB SR | 1506 Primary biliary cirrhosis |
| 1507 | UKBB SR | 1507 Haemochromatosis |
| 702 | ICD9 | 070.2 Viral hepatitis B with hepatic coma |
| 703 | ICD9 | 070.3 Viral hepatitis B without mention of hepatic coma |
| 704 | ICD9 | 070.4 Other specified viral hepatitis with hepatic coma |
| 705 | ICD9 | 070.5 Other specified viral hepatitis without mention of hepatic coma |
| 706 | ICD9 | 070.6 Unspecified viral hepatitis with hepatic coma |
| 709 | ICD9 | 070.9 Unspecified viral hepatitis without mention of hepatic coma |
| 2751 | ICD9 | 275.1 Wilson's disease |
| 27502 | ICD9 | 275.02 Hemochromatosis |
| 27761 | ICD9 | 277.61 Alpha-1-antitrypsin deficiency |
| 4530 | ICD9 | 453.0 Budd Chiari |
| NA | ICD9 | 571 Alcoholic fatty liver disease |
| 5710 | ICD9 | 571.0 Alcoholic fatty liver disease |
| 5711 | ICD9 | 571.1 Acute alcoholic hepatitis |
| 5712 | ICD9 | 571.2 Alcoholic cirrhosis of liver |
| 5713 | ICD9 | 571.3 Alcoholic liver damage unspecified |
| 5714 | ICD9 | 571.4 Chronic hepatitis unspecified (includes autoimmune hepatitis) |
| 5716 | ICD9 | 571.6 Biliary cirrhosis |
| NA | ICD10 | K70 Alcoholic liver disease |
| K700 | ICD10 | K70.0 Alcoholic fatty liver |
| K701 | ICD10 | K70.1 Alcoholic fatty liver |
| K702 | ICD10 | K70.2 Alcoholic fibrosis and sclerosis of liver |
| K703 | ICD10 | K70.3 Alcoholic cirrhosis of liver |
| K704 | ICD10 | K70.4 Alcoholic hepatic failure |
| K709 | ICD10 | K70.9 Alcoholic liver disease unspecified |
| K852 | ICD10 | K85.2 Alcohol-induced acute pancreatitis |
| K860 | ICD10 | K86.0 Alcohol-induced chronic pancreatitis |
| NA | ICD10 | B18 Chronic viral hepatitis |
| B180 | ICD10 | B18.0 Chronic viral hepatitis B with delta-agent |
| B181 | ICD10 | B18.1 Chronic viral hepatitis B without delta-agent |
| B182 | ICD10 | B18.2 Chronic viral hepatitis C |
| B188 | ICD10 | B18.8 Other chronic viral hepatitis |
| B189 | ICD10 | B18.9 Chronic viral hepatitis unspecified |
| NA | ICD10 | B19 Unspecified viral hepatitis |
| B190 | ICD10 | B19.0 Unspecified viral hepatitis with hepatic coma |
| NA | ICD10 | B19.1 Unspecified viral hepatitis B |
| NA | ICD10 | B19.2 Unspecified viral hepatitis C |
| B199 | ICD10 | B19.9 Unspecified viral hepatitis without hepatic coma |
| E830 | ICD10 | E83.0 Wilson's disease |
| E831 | ICD10 | E83.1 Hemochromatosis |
| E880 | ICD10 | E88.0 Alpha-1-antitrypsin deficiency |
| I820 | ICD10 | I82.0 Budd Chiari |
| NA | ICD10 | K71 Toxic liver disease |
| K710 | ICD10 | K71.0 Toxic liver disease with cholestasis |
| K711 | ICD10 | K71.1 Toxic liver disease with hepatic necrosis |
| K712 | ICD10 | K71.2 Toxic liver disease with acute hepatitis |
| K713 | ICD10 | K71.3 Toxic liver disease with chronic persistent hepatitis |
| K714 | ICD10 | K71.4 Toxic liver disease with chronic lobular hepatitis |
| K715 | ICD10 | K71.5 Toxic liver disease with chronic active hepatitis |
| K716 | ICD10 | K71.6 Toxic liver disease with hepatitis not elsewhere classified |
| K717 | ICD10 | K71.7 Toxic liver disease with fibrosis and cirrhosis of liver |
| K718 | ICD10 | K71.8 Toxic liver disease with other disorders of liver |
| K719 | ICD10 | K71.9 Toxic liver disease unspecified |
| K732 | ICD10 | K73.2 Chronic active hepatitis, not elsewhere classified |
| K739 | ICD10 | K73.9 Chronic hepatitis, unspecified |
| K743 | ICD10 | K74.3 Primary biliary cirrhosis |
| K744 | ICD10 | K74.4 Secondary biliary cirrhosis |
| K745 | ICD10 | K74.5 Biliary cirrhosis, unspecified |
| K754 | ICD10 | K75.4 Autoimmune hepatitis |
| K765 | ICD10 | K76.5 Hepatic veno-occlusive disease |
| K830 | ICD10 | K83.0 Primary sclerosing cholangitis |

| Supplementary Method Table 2. Codes used to identify participants with evidence of alcohol dependency | | |
| --- | --- | --- |
| **UKBB code** | **Code type** | **Code** |
| 1408 | UKBB SR | 1408 Alcohol dependency |
| NA | ICD9 | 291 Alcoholic psychoses |
| 2910 | ICD9 | 291.0 Delerium tremens |
| 2911 | ICD9 | 291.1 Korsakov's psychosis alcoholic |
| 2912 | ICD9 | 291.2 Other alcoholic dementia |
| 2913 | ICD9 | 291.3 Other alcoholic hallucinations |
| 2914 | ICD9 | 291.4 Pathological drunkenness |
| 2915 | ICD9 | 291.5 Alcoholic jealousy |
| 2918 | ICD9 | 291.8 Other specified alcoholic psychoses |
| 2919 | ICD9 | 291.9 Alcoholic psychoses unspecified |
| NA | ICD9 | 303 Alcohol dependence syndrome |
| 3039 | ICD9 | 303.9 Other and unspecified alcohol dependence |
| 3050 | ICD9 | 305.0 Nondependent alcohol abuse |
| 3575 | ICD9 | 357.5 Alcoholic polyneuropathy |
| 4255 | ICD9 | 425.5 Alcoholic cardiomyopathy |
| 5353 | ICD9 | 535.3 Alcoholic gastritis |
| 9800 | ICD9 | 980.1 Toxic effect of ethyl alcohol |
| 9809 | ICD9 | 980.9 Toxic effect of unspecified alcohol |
| E244 | ICD10 | E24.4 Alcohol-induced pseudo-Cushing's syndrome |
| E512 | ICD10 | E51.2 Wernicke's encephalopathy |
| F101 | ICD-10 | F10.1 Harmful use |
| F102 | ICD-10 | F10.2 Dependence syndrome |
| F103 | ICD-10 | F10.3 Withdrawal state |
| F104 | ICD-10 | F10.4 Withdrawal state with delirium |
| F105 | ICD-10 | F10.5 Psychotic disorder |
| F106 | ICD-10 | F10.6 Amnesic syndrome |
| F107 | ICD-10 | F10.7 Residual and late-onset psychotic disorder |
| F108 | ICD-10 | F10.8 Other mental and behavioural disorders |
| F109 | ICD-10 | F10.9 Unspecified mental and behavioural disorder |
| G312 | ICD10 | G31.2 Degeneration of the nervous system due to alcohol |
| G621 | ICD10 | G62.1 Alcoholic polyneuropathy |
| G721 | ICD10 | G72.1 Alcoholic myopathy |
| I426 | ICD10 | I42.6 Alcoholic cardiomyopathy |
| K292 | ICD10 | K29.2 Alcoholic gastritis |
| T510 | ICD10 | T51.0 Toxic effect of ethanol |
| T519 | ICD10 | T51.9 Toxic effect of unspecified alcohol |
| X6509 | ICD-10 | X65.09 Intentional self-poisoning by and exposure to alcohol, Home, During unspecified activity |
| Y573 | ICD10 | Y57.3 Adverse effects in therapeutic use: alcohol deterrents |
| Z509 | ICD10 | Z50.2 Alcohol rehabilitation |
| Z714 | ICD10 | Z71.4 Alcohol abuse counselling and surveillance |

| Supplementary Method Table 3. Codes used to identify those with evidence of intrinsic causes of renal disease | |
| --- | --- |
| **UKBB Code** | **Code** |
| N00 | N00 Acute nephritic syndrome |
| N000 | N00.0 Minor glomerular abnormality |
| N001 | N00.1 Focal and segmental glomerular lesions |
| N002 | N00.2 Diffuse membranous glomerulonephritis |
| N003 | N00.3 Diffuse mesangial proliferative glomerulonephritis |
| N004 | N00.4 Diffuse endocapillary proliferative glomerulonephritis |
| N005 | N00.5 Diffuse mesangiocapillary glomerulonephritis |
| N006 | N00.6 Dense deposit disease |
| N007 | N00.7 Diffuse crescentic glomerulonephritis |
| N008 | N00.8 Other |
| N009 | N00.9 Unspecified |
| N01 | N01 Rapidly progressive nephritic syndrome |
| N010 | N01.0 Minor glomerular abnormality |
| N011 | N01.1 Focal and segmental glomerular lesions |
| N012 | N01.2 Diffuse membranous glomerulonephritis |
| N013 | N01.3 Diffuse mesangial proliferative glomerulonephritis |
| N014 | N01.4 Diffuse endocapillary proliferative glomerulonephritis |
| N015 | N01.5 Diffuse mesangiocapillary glomerulonephritis |
| N016 | N01.6 Dense deposit disease |
| N017 | N01.7 Diffuse crescentic glomerulonephritis |
| N018 | N01.8 Other |
| N019 | N01.9 Unspecified |
| N02 | N02 Recurrent and persistent haematuria |
| N020 | N02.0 Minor glomerular abnormality |
| N021 | N02.1 Focal and segmental glomerular lesions |
| N022 | N02.2 Diffuse membranous glomerulonephritis |
| N023 | N02.3 Diffuse mesangial proliferative glomerulonephritis |
| N024 | N02.4 Diffuse endocapillary proliferative glomerulonephritis |
| N025 | N02.5 Diffuse mesangiocapillary glomerulonephritis |
| N026 | N02.6 Dense deposit disease |
| N027 | N02.7 Diffuse crescentic glomerulonephritis |
| N028 | N02.8 Other |
| N029 | N02.9 Unspecified |
| N03 | N03 Chronic nephritic syndrome |
| N030 | N03.0 Minor glomerular abnormality |
| N031 | N03.1 Focal and segmental glomerular lesions |
| N032 | N03.2 Diffuse membranous glomerulonephritis |
| N033 | N03.3 Diffuse mesangial proliferative glomerulonephritis |
| N034 | N03.4 Diffuse endocapillary proliferative glomerulonephritis |
| N035 | N03.5 Diffuse mesangiocapillary glomerulonephritis |
| N036 | N03.6 Dense deposit disease |
| N037 | N03.7 Diffuse crescentic glomerulonephritis |
| N038 | N03.8 Other |
| N039 | N03.9 Unspecified |
| N04 | N04 Nephrotic syndrome |
| N040 | N04.0 Minor glomerular abnormality |
| N041 | N04.1 Focal and segmental glomerular lesions |
| N042 | N04.2 Diffuse membranous glomerulonephritis |
| N043 | N04.3 Diffuse mesangial proliferative glomerulonephritis |
| N044 | N04.4 Diffuse endocapillary proliferative glomerulonephritis |
| N045 | N04.5 Diffuse mesangiocapillary glomerulonephritis |
| N046 | N04.6 Dense deposit disease |
| N047 | N04.7 Diffuse crescentic glomerulonephritis |
| N048 | N04.8 Other |
| N049 | N04.9 Unspecified |
| N05 | N05 Unspecified nephritic syndrome |
| N050 | N05.0 Minor glomerular abnormality |
| N051 | N05.1 Focal and segmental glomerular lesions |
| N052 | N05.2 Diffuse membranous glomerulonephritis |
| N053 | N05.3 Diffuse mesangial proliferative glomerulonephritis |
| N054 | N05.4 Diffuse endocapillary proliferative glomerulonephritis |
| N055 | N05.5 Diffuse mesangiocapillary glomerulonephritis |
| N056 | N05.6 Dense deposit disease |
| N057 | N05.7 Diffuse crescentic glomerulonephritis |
| N058 | N05.8 Other |
| N059 | N05.9 Unspecified |
| N06 | N06 Isolated proteinuria with specified morphological lesion |
| N060 | N06.0 Minor glomerular abnormality |
| N061 | N06.1 Focal and segmental glomerular lesions |
| N062 | N06.2 Diffuse membranous glomerulonephritis |
| N063 | N06.3 Diffuse mesangial proliferative glomerulonephritis |
| N064 | N06.4 Diffuse endocapillary proliferative glomerulonephritis |
| N065 | N06.5 Diffuse mesangiocapillary glomerulonephritis |
| N066 | N06.6 Dense deposit disease |
| N067 | N06.7 Diffuse crescentic glomerulonephritis |
| N068 | N06.8 Other |
| N069 | N06.9 Unspecified |
| N07 | N07 Hereditary nephropathy, not elsewhere classified |
| N070 | N07.0 Minor glomerular abnormality |
| N071 | N07.1 Focal and segmental glomerular lesions |
| N072 | N07.2 Diffuse membranous glomerulonephritis |
| N073 | N07.3 Diffuse mesangial proliferative glomerulonephritis |
| N074 | N07.4 Diffuse endocapillary proliferative glomerulonephritis |
| N075 | N07.5 Diffuse mesangiocapillary glomerulonephritis |
| N076 | N07.6 Dense deposit disease |
| N077 | N07.7 Diffuse crescentic glomerulonephritis |
| N078 | N07.8 Other |
| N079 | N07.9 Unspecified |
| N08 | N08 Glomerular disorders in diseases classified elsewhere |
| N080 | N08.0 Glomerular disorders in infectious and parasitic diseases classified elsewhere |
| N081 | N08.1 Glomerular disorders in neoplastic diseases |
| N082 | N08.2 Glomerular disorders in blood diseases and disorders involving the immune mechanism |
| N083 | N08.3 Glomerular disorders in diabetes mellitus |
| N084 | N08.4 Glomerular disorders in other endocrine, nutritional and metabolic diseases |
| N085 | N08.5 Glomerular disorders in systemic connective tissue disorders |
| N088 | N08.8 Glomerular disorders in other diseases classified elsewhere |
| N10 | N10 Acute tubulo-interstitial nephritis |
| N11 | N11 Chronic tubulo-interstitial nephritis |
| N110 | N11.0 Nonobstructive reflux-associated chronic pyelonephritis |
| N111 | N11.1 Chronic obstructive pyelonephritis |
| N118 | N11.8 Other chronic tubulo-interstitial nephritis |
| N119 | N11.9 Chronic tubulo-interstitial nephritis, unspecified |
| N12 | N12 Tubulo-interstitial nephritis, not specified as acute or chronic |
| N13 | N13 Obstructive and reflux uropathy |
| N130 | N13.0 Hydronephrosis with ureteropelvic junction obstruction |
| N131 | N13.1 Hydronephrosis with ureteral stricture, not elsewhere classified |
| N132 | N13.2 Hydronephrosis with renal and ureteral calculous obstruction |
| N133 | N13.3 Other and unspecified hydronephrosis |
| N134 | N13.4 Hydroureter |
| N135 | N13.5 Kinking and stricture of ureter without hydronephrosis |
| N136 | N13.6 Pyonephrosis |
| N137 | N13.7 Vesicoureteral-reflux-associated uropathy |
| N138 | N13.8 Other obstructive and reflux uropathy |
| N139 | N13.9 Obstructive and reflux uropathy, unspecified |
| N14 | N14 Drug- and heavy-metal-induced tubulo-interstitial and tubular conditions |
| N140 | N14.0 Analgesic nephropathy |
| N141 | N14.1 Nephropathy induced by other drugs, medicaments and biological substances |
| N142 | N14.2 Nephropathy induced by unspecified drug, medicament or biological substance |
| N143 | N14.3 Nephropathy induced by heavy metals |
| N144 | N14.4 Toxic nephropathy, not elsewhere classified |
| N15 | N15 Other renal tubulo-interstitial diseases |
| N150 | N15.0 Balkan nephropathy |
| N151 | N15.1 Renal and perinephric abscess |
| N158 | N15.8 Other specified renal tubulo-interstitial diseases |
| N159 | N15.9 Renal tubulo-interstitial disease, unspecified |
| N16 | N16 Renal tubulo-interstitial disorders in diseases classified elsewhere |
| N160 | N16.0 Renal tubulo-interstitial disorders in infectious and parasitic diseases classified elsewhere |
| N161 | N16.1 Renal tubulo-interstitial disorders in neoplastic diseases |
| N162 | N16.2 Renal tubulo-interstitial disorders in blood diseases and disorders involving the immune mechanism |
| N163 | N16.3 Renal tubulo-interstitial disorders in metabolic diseases |
| N164 | N16.4 Renal tubulo-interstitial disorders in systemic connective tissue disorders |
| N165 | N16.5 Renal tubulo-interstitial disorders in transplant rejection |
| N168 | N16.8 Renal tubulo-interstitial disorders in other diseases classified elsewhere |
| N17 | N17 Acute renal failure |
| N170 | N17.0 Acute renal failure with tubular necrosis |
| N171 | N17.1 Acute renal failure with acute cortical necrosis |
| N172 | N17.2 Acute renal failure with medullary necrosis |
| N178 | N17.8 Other acute renal failure |
| N179 | N17.9 Acute renal failure, unspecified |
| N28 | N28 Other disorders of kidney and ureter, not elsewhere classified |
| N280 | N28.0 Ischaemia and infarction of kidney |
| N281 | N28.1 Cyst of kidney, acquired |
| N288 | N28.8 Other specified disorders of kidney and ureter |
| N289 | N28.9 Disorder of kidney and ureter, unspecified |
| N29 | N29 Other disorders of kidney and ureter in diseases classified elsewhere |
| N290 | N29.0 Late syphilis of kidney |
| N291 | N29.1 Other disorders of kidney and ureter in infectious and parasitic diseases classified elsewhere |
| N298 | N29.8 Other disorders of kidney and ureter in other diseases classified elsewhere |

| Supplementary Method Table 4. ICD-9/10 codes used to identify participants with SLD | | |
| --- | --- | --- |
| **UKBB code** | **Code type** | **Code** |
| 5718 | ICD-9 | 571.8 Other chronic non-alcoholic liver disease |
| K758 | ICD-10 | K75.8 Other specified inflammatory liver diseases |
| K760 | ICD-10 | K76.0 Fatty (change of) liver, not elsewhere classified |

| Supplementary Method Table 5. Variables and criteria used to identify participants with metabolic syndrome traits | | |
| --- | --- | --- |
| **Metabolic syndrome trait(s)** | **Variables used** | **Thresholds used** |
| Central obesity | Waist circumference (data field: 48). | >94 cm (men)  Or  >80 cm (women) |
| Dysglycaemia/T2D | Hemoglobin A1c (data field: 30750)  Or  ICD9/10 or self-reported codes or use of anti-hyperglycemic treatments (Supplementary Table 5 & 6)). After excluding the presence of T1DM or gestational diabetes (Supplementary Table 7). | ≥39 mmol/mol |
| Increased blood pressure | Diastolic (data field: 4080) and systolic (data field: 4079) blood pressures  Or  ICD9/10 or self-reported codes or use of anti-hypertension treatment (Supplementary Table 8 & 9)). | ≥85 mmHg (diastolic)  Or  ≥130 mmHg (systolic) |
| High triglycerides | Triglyceride concentrations (data field: 30870)  Or  ICD9/10 or self-reported codes or use of lipid-lowering treatment (Supplementary Table 10 & 11). | ≥1.70 mmol/l |
| Low HDL cholesterol | HDL concentrations (data field: 30760)  Or  ICD9/10 or self-reported codes or use of lipid-lowering treatment (Supplementary Table 10 & 11). | (≤1.0 mmol/l (men)  Or  ≤1.3 mmol/l (women) |
| Those participants with ≥3 traits were identified as having MetS.  Thresholds are in accordance with those provided by Alberti et al. ^1^ and as outlined in the MASLD multi-society Delphi consensus statement published in 2023 ^2^. | | |

| **Supplementary** **Method Table 6. ICD10 and self-report codes used to identify participants with T2D** | | |
| --- | --- | --- |
| UKBB code | **Code type** | **Code** |
| NA | ICD10 | E10 Insulin-dependent diabetes mellitus |
| E100 | ICD10 | E10.0 With coma |
| E101 | ICD10 | E10.1 With ketoacidosis |
| E102 | ICD10 | E10.2 With renal complications |
| E103 | ICD10 | E10.3 With ophthalmic complications |
| E104 | ICD10 | E10.4 With neurological complications |
| E105 | ICD10 | E10.5 With peripheral circulatory complications |
| E106 | ICD10 | E10.6 With other specified complications |
| E107 | ICD10 | E10.7 With multiple complications |
| E108 | ICD10 | E10.8 With unspecified complications |
| E109 | ICD10 | E10.9 Without complications |
| NA | ICD10 | E11 Non-insulin-dependent diabetes mellitus |
| E110 | ICD10 | E11.0 With coma |
| E111 | ICD10 | E11.1 With ketoacidosis |
| E112 | ICD10 | E11.2 With renal complications |
| E113 | ICD10 | E11.3 With ophthalmic complications |
| E114 | ICD10 | E11.4 With neurological complications |
| E115 | ICD10 | E11.5 With peripheral circulatory complications |
| E116 | ICD10 | E11.6 With other specified complications |
| E117 | ICD10 | E11.7 With multiple complications |
| E118 | ICD10 | E11.8 With unspecified complications |
| E119 | ICD10 | E11.9 Without complications |
| 1220 | UKBB SR | 1220 Diabetes |
| 1223 | UKBB SR | 1223 Type 2 diabetes |

| **Supplementary Method Table 7. Anti-hyperglycaemia medication codes used to identify those with T2D** | |
| --- | --- |
| Insulin | |
| sn1140883066 | insulin product |
| Metformin | |
| 1141189094 | avandamet 1mg / 500mg tablet |
| 1140921964 | glucamet 500 tablet |
| 1140874686 | glucophage 500mg tablet |
| 1140884600 | Metformin |
| Sulphonylurea | |
| 1140857584 | acetohexamide |
| 1141156984 | amaryl 1mg tablet |
| 1140874740 | calabren 2.5mg tablet |
| 1140874706 | chlorpropamide |
| 1140874724 | daonil 5mg tablet |
| 1140874736 | diabetamide 2.5mg tablet |
| 1141169504 | diaglyk 80mg tablet |
| 1140874712 | diabinese 100mg tablet |
| 1140874746 | diamicron 80mg tablet |
| 1140857586 | dimelor 500mg tablet |
| 1140874728 | euglucon 2.5mg tablet |
| 1140874650 | glibenese 5mg tablet |
| 1140874718 | Glibenclamide |
| 1140874744 | Gliclazide |
| 1141152590 | Glimepiride |
| 1140874646 | Glipizide |
| 1141157284 | glipizide product |
| 1140874658 | Gliquidone |
| 1140874660 | glurenorm 30mg tablet |
| 1140874678 | glyconon 500mg tablet |
| 1140874716 | glymese 250mg tablet |
| 1140857590 | libanil 2.5mg tablet |
| 1140874732 | malix 2.5mg tablet |
| 1140874652 | minodiab 2.5mg tablet |
| 1140874690 | orabet 500mg tablet |
| 1140857506 | pramidex 500mg tablet |
| 1140874680 | rastinon 500mg tablet |
| 1140874726 | semi-daonil 2.5mg tablet |
| 1140874666 | tolanase 100mg tablet |
| 1140874664 | Tolazamide |
| 1140874674 | Tolbutamide |
| Thiazolidinedione | |
| 1141171652 | actos 15mg tablet |
| 1141177606 | avandia 4mg tablet |
| 1141171646 | Pioglitazone |
| 1141153262 | romozin 200mg tablet |
| 1141177600 | Rosiglitazone |
| 1141189090 | rosiglitazone 1mg / metformin 500mg tablet |
| 1141153254 | Troglitazone |
| Sulphonamide | |
| 1140857500 | Glymidine |
| 1140857502 | gondafon 500mg tablet |
| Meglitinide | |
| 1141173882 | Nateglinide |
| 1141168668 | novonorm 0.5mg tablet |
| 1141168660 | Repaglinide |
| 1141173786 | starlix 60mg tablet |
| Alpha glucosidases inhibitor | |
| 1140868902 | Acarbose |
| 1140868908 | glucobay 50mg tablet |
| Guar preparations | |
| 1140857508 | glucotard 5g/sachet mini-tablet |
| 1140857510 | lejguar 90% granules |

| **Supplementary Method Table 8. Codes used to exclude participants with T1DM or gestational diabetes** | | |
| --- | --- | --- |
| UKBB code | **Code type** | **Code** |
| 1221 | UKBB SR | 1221 Gestational diabetes |
| 1222 | UKBB SR | 1222 Type 1 diabetes |

| **Supplementary Method Table 9. Codes used to identify participants with hypertension** | | |
| --- | --- | --- |
| UKBB code | **Code type** | **Code** |
| 1065 | UKBB SR | Hypertension |
| 1072 | UKBB SR | Essential hypertension |
| NA | ICD9 | 401 Essential hypertension |
| 4010 | ICD9 | 401.0 Essential hypertension, specified as malignant |
| 4011 | ICD9 | 401.1 Essential hypertension, specified as benign |
| 4019 | ICD9 | 401.9 Essential hypertension, not specified as malignant or benign |
| NA | ICD9 | 402 Hypertensive heart disease |
| 4020 | ICD9 | 402.0 Hypertensive heart disease, specified as malignant |
| 4021 | ICD9 | 402.1 Hypertensive heart disease, specified as benign |
| 4029 | ICD9 | 402.9 Hypertensive heart disease, not specified as malignant or benign |
| NA | ICD9 | 403 Hypertensive renal disease |
| 4030 | ICD9 | 403.0 Hypertensive renal disease, specified as malignant |
| 4031 | ICD9 | 403.1 Hypertensive renal disease, specified as benign |
| 4039 | ICD9 | 403.9 Hypertensive renal disease, not specified as malignant or benign |
| NA | ICD9 | 404 Hypertensive heart and renal disease |
| 4040 | ICD9 | 404.0 Hypertensive heart and renal disease, specified as malignant |
| 4041 | ICD9 | 404.1 Hypertensive heart and renal disease, specified as benign |
| 4049 | ICD9 | 404.9 Hypertensive heart and renal disease, not specified as malignant or benign |
| 4372 | ICD9 | 437.2 Hypertensive encephalopathy |
| I10 | ICD10 | I10 Essential (primary) hypertension |
| NA | ICD10 | I11 Hypertensive heart disease |
| I110 | ICD10 | I11.0 Hypertensive heart disease with (congestive) heart failure |
| I119 | ICD10 | I11.9 Hypertensive heart disease without (congestive) heart failure |
| NA | ICD10 | I12 Hypertensive renal disease |
| I120 | ICD10 | I12.0 Hypertensive renal disease with renal failure |
| I129 | ICD10 | I12.9 Hypertensive renal disease without renal failure |
| NA | ICD10 | I13 Hypertensive heart and renal disease |
| I130 | ICD10 | I13.0 Hypertensive heart and renal disease with (congestive) heart failure |
| I131 | ICD10 | I13.1 Hypertensive heart and renal disease with renal failure |
| I132 | ICD10 | I13.2 Hypertensive heart and renal disease with both (congestive) heart failure and renal failure |
| I139 | ICD10 | I13.9 Hypertensive heart and renal disease, unspecified |
| I674 | ICD10 | I67.4 Hypertensive encephalopathy |

| **Supplementary Method Table 10. Anti-hypertension medication codes used to identify participants with hypertension** | |
| --- | --- |
| Code | **Medication** |
| 1140866724 | Acebutolol |
| 1140860422 | acebutolol+hydrochlorothiazide 200mg/12.5mg tablet |
| 1140881706 | accupro 5mg tablet |
| 1140860736 | accuretic tablet |
| 1140860752 | acepril 12.5mg tablet |
| 1140861090 | adalat 5mg capsule |
| 1140881702 | adalate 10mg capsule |
| 1140923572 | adipine mr 10 m/r tablet |
| 1140861138 | adizem-60 m/r tablet |
| 1140926780 | adizem-xl plus m/r capsule |
| 1140866396 | aldactide 25 tablet |
| 1140866244 | aldactone 25mg tablet |
| 1140851342 | aluzine 20mg tablet |
| 1140927174 | amilamont 5mg/ml s/f oral solution |
| 1140866354 | amilmaxco 5/50 tablet |
| 1140888512 | Amiloride |
| 1140866422 | amiloride hcl+cyclopenthiazide 2.5mg/250micrograms tablet |
| 1140866426 | amiloride hydrochloride+bumetanide 5mg/1mg tablet |
| 1140866222 | amilospare 5mg tablet |
| 1140879802 | Amlodipine |
| 1141200400 | amlostin 5mg tablet |
| 1140866704 | angilol 10mg tablet |
| 1140861110 | angiopine 5mg capsule |
| 1140861136 | angiozem 60mg m/r tablet |
| 1140917428 | angitil sr 90 m/r capsule |
| 1140888578 | antihypertensive |
| 1140864410 | antipressan 25mg tablet |
| 1140866128 | aprinox 2.5mg tablet |
| 1140866764 | apsolol 10mg tablet |
| 1140860212 | apsolox 20mg tablet |
| 1140860180 | arbralene 50mg tablet |
| 1140866212 | arelix 6mg capsule |
| 1140922930 | atenix 25mg tablet |
| 1140860348 | atenixco 50mg/12.5mg tablet |
| 1140866738 | Atenolol |
| 1141146126 | atenolol+bendrofluazide |
| 1141194810 | atenolol+bendroflumethiazide |
| 1141180778 | atenolol+chlortalidone |
| 1141146124 | atenolol+chlorthalidone |
| 1141146128 | atenolol+co-amilozide |
| 1140860426 | atenolol+nifedipine 50mg/20mg m/r capsule |
| 1140866086 | baycaron 25mg tablet |
| 1140851556 | bedranol 10mg tablet |
| 1140866122 | Bendrofluazide |
| 1140866450 | bendrofluazide+potassium 2.5mg/7.7mmol m/r tablet |
| 1141194794 | bendroflumethiazide |
| 1141194800 | bendroflumethiazide+potassium 2.5mg/7.7mmol m/r tablet |
| 1140866226 | berkamil 5mg tablet |
| 1140866546 | berkatens 40mg tablet |
| 1140866784 | berkolol 10mg tablet |
| 1140866132 | berkozide 2.5mg tablet |
| 1140860356 | beta-adalat capsule |
| 1140866782 | beta-prograne 160mg m/r capsule |
| 1140851492 | betadren 5mg tablet |
| 1140866778 | betadur cr 160mg m/r capsule |
| 1140860266 | betaloc 50mg tablet |
| 1141168964 | betinex 1mg tablet |
| 1141175224 | bi-carzem sr 60mg m/r capsule |
| 1141184324 | bipranix 5mg tablet |
| 1140879760 | Bisoprolol |
| 1140864950 | bisoprolol fumarate+hydrochlorothiazide 10mg/6.25mg tablet |
| 1140861130 | britiazim 60mg m/r tablet |
| 1140866280 | Bumetanide |
| 1140866448 | bumetanide+potassium 500micrograms/7.7mmol m/r tablet |
| 1140866282 | burinex 1mg tablet |
| 1140866356 | burinex a tablet |
| 1140866438 | burinex k m/r tablet |
| 1140910442 | bzt-bendrofluazide |
| 1141187094 | cabren 2.5mg m/r tablet |
| 1140916930 | calanif 5mg capsule |
| 1141153454 | calazem 60mg m/r tablet |
| 1141173766 | calchan mr 10mg m/r tablet |
| 1140851730 | calcicard 60mg tablet |
| 1140861106 | calcilat 10mg capsule |
| 1140860758 | capoten 12.5mg tablet |
| 1140860750 | Captopril |
| 1140860764 | captopril+hydrochlorothiazide 25mg/12.5mg tablet |
| 1140864910 | carace 10 plus tablet |
| 1140860706 | carace 2.5mg tablet |
| 1140861176 | cardene 20mg capsule |
| 1141171152 | cardicor 1.25mg tablet |
| 1140927934 | cardilate mr 10mg m/r tablet |
| 1140866712 | cardinol 10mg tablet |
| 1141199858 | cardioplen xl 5mg m/r tablet |
| 1140909368 | Carvedilol |
| 1140879762 | Celiprolol |
| 1140851332 | centyl 2.5mg tablet |
| 1140866440 | centyl k m/r tablet |
| 1140866138 | Chlorothiazide |
| 1140909706 | Chlortalidone |
| 1140866144 | Chlorthalidone |
| 1140864202 | chlorthalidone tablet+potassium m/r tablet 25mg/6.7mmol pack |
| 1140860882 | Cilazapril |
| 1140923276 | co-amilozide |
| 1140860386 | co-betaloc tablet |
| 1140923282 | co-flumactone |
| 1140923336 | co-tenidone |
| 1140861120 | coracten sr 10mg m/r capsule |
| 1140866554 | cordilox 40mg tablet |
| 1140860194 | corgard 40mg tablet |
| 1141166752 | coroday mr 20mg m/r tablet |
| 1140860802 | coversyl 2mg tablet |
| 1140866156 | cyclopenthiazide |
| 1140866340 | delvas tablet |
| 1140851418 | diatensec 50mg tablet |
| 1141157136 | dilcardia sr 60mg m/r capsule |
| 1140879806 | Diltiazem |
| 1140926778 | diltiazem hcl+hydrochlorothiazide 150mg/12.5mg m/r capsule |
| 1140861166 | dilzem sr 60mg long acting m/r capsule |
| 1141185444 | disogram sr 60mg m/r capsule |
| 1140851400 | diuresal 40mg tablet |
| 1140866110 | diurexan 20mg tablet |
| 1140866182 | dryptal 40mg tablet |
| 1140866402 | dyazide tablet |
| 1140866390 | dytac 50mg capsule |
| 1141150328 | ecopace 12.5mg tablet |
| 1140866202 | edecrin 50mg tablet |
| 1140860492 | emcor 10mg tablet |
| 1140888552 | Enalapril |
| 1140860790 | enalapril maleate+hydrochlorothiazide 20mg/12.5mg tablet |
| 1140851338 | enduron 5mg tablet |
| 1141201244 | Eplerenone |
| 1140866164 | esidrex 25mg tablet |
| 1140851362 | esidrex k tablet |
| 1140866200 | ethacrynic acid |
| 1140866206 | ethacrynic acid 50mg tablet |
| 1141157184 | ethacrynic acid product |
| 1141169096 | ethimil mr 240 m/r tablet |
| 1141168498 | eucardic 3.125 tablet |
| 1141188836 | felendil xl 5mg m/r tablet |
| 1140888646 | Felodipine |
| 1141165470 | felodipine+ramipril |
| 1141188576 | felogen xl 5mg m/r tablet |
| 1141188152 | felotens xl 5mg m/r tablet |
| 1141145870 | fortipine la40 m/r tablet |
| 1140888556 | Fosinopril |
| 1140866192 | froop 40mg tablet |
| 1141167108 | froop co 5mg/40mg tablet |
| 1140866418 | fru-co tablet |
| 1140851414 | frumax 40mg tablet |
| 1140866406 | frumil tablet |
| 1140866116 | Frusemide |
| 1140866408 | frusene tablet |
| 1140851412 | frusetic 40mg tablet |
| 1140866194 | frusid 40mg tablet |
| 1141169088 | frusol 20mg/5ml s/f oral solution |
| 1140909708 | Furosemide |
| 1141195258 | furosemide+potassium 20mg/10mmol m/r tablet |
| 1140866484 | geangin 40mg tablet |
| 1141152600 | genalat retard 10mg m/r tablet |
| 1140860912 | gopten 500micrograms capsule |
| 1140866802 | half beta-prograne 80mg m/r capsule |
| 1141152076 | half propanix la 80mg m/r capsule |
| 1141156754 | half propatard la 80mg m/r capsule |
| 1140866460 | half securon sr 120mg m/r tablet |
| 1140866798 | half-betadur cr 80mg m/r capsule |
| 1140866800 | half-inderal la 80mg m/r capsule |
| 1141180238 | horizem sr 90mg m/r capsule |
| 1140866168 | hydrosaluric 25mg tablet |
| 1140851364 | hygroton k tablet combination pack |
| 1141151382 | hypapril 12.5mg tablet |
| 1140851432 | hypertane-50 tablet |
| 1141167758 | hyteneze 12.5 tablet |
| 1141164148 | imidapril hydrochloride |
| 1140866078 | Indapamide |
| 1140866804 | inderal 10mg tablet |
| 1140860776 | innovace 2.5mg tablet |
| 1140860784 | innozide tablet |
| 1141201250 | inspra 25mg tablet |
| 1141172682 | irbesartan+hydrochlorothiazide 150mg/12.5mg tablet |
| 1140861190 | Isradipine |
| 1140866410 | kalspare tablet |
| 1141150560 | kaplon 12.5mg tablet |
| 1141188920 | keloc sr 5mg m/r tablet |
| 1140923618 | kentiazem 60mg m/r capsule |
| 1141187962 | kentipine mr 10mg m/r tablet |
| 1140879824 | Labetalol |
| 1140860244 | labrocol 100mg tablet |
| 1140861276 | Lacidipine |
| 1140851576 | laracor 20mg tablet |
| 1140851420 | laractone 25mg tablet |
| 1140866412 | lasilactone capsule |
| 1140866248 | lasix 20mg tablet |
| 1140866334 | lasoride tablet |
| 1141153026 | Lercanidipine |
| 1140860696 | Lisinopril |
| 1140864952 | lisinopril+hydrochlorothiazide 10mg/12.5mg tablet |
| 1141199940 | lopace 2.5mg capsule |
| 1140917076 | lopranol la 160mg m/r capsule |
| 1140860274 | lopresor 50mg tablet |
| 1140860402 | lopresoretic tablet |
| 1140866084 | Mefruside |
| 1140860278 | mepranix 50mg tablet |
| 1140866094 | metenix-5 tablet |
| 1140866090 | methyclothiazide |
| 1140875808 | Metipranolol |
| 1140860828 | Metirosine |
| 1140866092 | Metolazone |
| 1140879818 | Metoprolol |
| 1140860308 | metoprolol tartrate+chlorthalidone 100mg/12.5mg tablet |
| 1140860404 | metoprolol tartrate+hydrochlorothiazide 100mg/12.5mg tablet |
| 1140851522 | metoros 95mg tablet |
| 1141153394 | Mibefradil |
| 1140866220 | midamor 5mg tablet |
| 1140866416 | moduret 25 tablet |
| 1140866420 | moduretic tablet |
| 1140923712 | Moexipril |
| 1140860434 | monocor 5mg tablet |
| 1140864176 | monozide 10 tablet |
| 1140861282 | motens 2mg tablet |
| 1140860192 | Nadolol |
| 1140860312 | nadolol+bendrofluazide 40mg/5mg tablet |
| 1140860316 | nadolol+bendrofluazide 80mg/5mg tablet |
| 1141194804 | nadolol+bendroflumethiazide 40mg/5mg tablet |
| 1141146378 | natrilix sr 1.5mg m/r tablet |
| 1140866158 | navidrex 500mcg tablet |
| 1140851368 | navidrex-k tablet |
| 1140866352 | navispare tablet |
| 1141164280 | nebilet 5mg tablet |
| 1141164276 | Nebivolol |
| 1140888918 | neo-bendromax 2.5mg tablet |
| 1140866136 | neo-naclex 5mg tablet |
| 1140866446 | neo-naclex k m/r tablet |
| 1141200782 | neofel xl 5mg m/r tablet |
| 1140866104 | nephril 1mg tablet |
| 1140879810 | Nicardipine |
| 1140861088 | Nifedipine |
| 1141157140 | nifedipress mr 10 m/r tablet |
| 1141150538 | nifedotard 20mr m/r tablet |
| 1140911088 | nifelease 20mg m/r tablet |
| 1140861114 | nifensar xl 20mg m/r tablet |
| 1141169730 | nifopress retard 20mg m/r tablet |
| 1140872568 | Nimodipine |
| 1140926966 | nimodrel mr 10 m/r tablet |
| 1140872472 | nimotop 30mg tablet |
| 1140888922 | nindaxa 2.5mg tablet |
| 1140928226 | Nisoldipine |
| 1141162546 | nivaten retard 10mg m/r tablet |
| 1140860918 | odrik 500micrograms capsule |
| 1141156656 | optil 60mg m/r tablet |
| 1140917068 | opumide 2.5mg tablet |
| 1140879830 | Oxprenolol |
| 1140860230 | oxyprenix sr 160mg m/r tablet |
| 1140851484 | paritane 20mg tablet |
| 1140868036 | parmid 10mg tablet |
| 1141201814 | parmid xl 5mg m/r tablet |
| 1140879834 | Penbutolol |
| 1140860320 | penbutolol sulphate+furosemide 40mg/20mg tablet |
| 1140923718 | perdix 7.5mg tablet |
| 1140888560 | Perindopril |
| 1141180592 | perindopril+indapamide |
| 1140860292 | Pindolol |
| 1140860322 | pindolol+clopamide 10mg/5mg tablet |
| 1140866210 | Piretanide |
| 1140928212 | plendil 2.5mg m/r tablet |
| 1140866102 | Polythiazide |
| 1141153400 | posicor 50mg tablet |
| 1141170870 | pralenal 2.5mg tablet |
| 1140861194 | prescal 2.5mg tablet |
| 1140910614 | Prindolol |
| 1140866766 | propanix 10mg tablet |
| 1141156808 | propatard la 160mg m/r capsule |
| 1140879842 | Propranolol |
| 1140860418 | propranolol hydrochloride+bendrofluazide 80mg/2.5mg capsule |
| 1140860738 | quinalapril+hydrochlorothiazide 10mg/12.5mg tablet |
| 1140860728 | Quinapril |
| 1140860806 | Ramipril |
| 1141200698 | ranace 1.25mg capsule |
| 1141187056 | ranvera mr 240mg m/r tablet |
| 1141187048 | rapranol sr 80mg m/r capsule |
| 1140881712 | renitec 5mg tablet |
| 1140866262 | rusyde 20mg tablet |
| 1140866726 | sectral 100mg capsule |
| 1140866466 | securon 40mg tablet |
| 1140851660 | serpasil-esidrex tablet |
| 1141150500 | slofedipine 20mg m/r tablet |
| 1140916730 | sloprolol 80mg m/r capsule |
| 1140911698 | slozem 120mg m/r capsule |
| 1140860318 | sotazide tablet |
| 1140866308 | spiretic 25mg tablet |
| 1140866312 | spiroctan 25mg tablet |
| 1140866318 | spirolone 25mg tablet |
| 1140866236 | Spironolactone |
| 1140851508 | spiroprop tablet |
| 1140866306 | spirospare 25mg tablet |
| 1140860878 | staril 10mg tablet |
| 1140851430 | synuretic tablet |
| 1140928234 | syscor mr 10mg m/r tablet |
| 1141164154 | tanatril 5mg tablet |
| 1141153316 | tarka 2mg/180mg m/r capsule |
| 1141187788 | telmisartan+hydrochlorothiazide 40mg/12.5mg tablet |
| 1140860358 | tenif capsule |
| 1140866756 | tenormin 25 tablet |
| 1140927940 | tensipine mr 10 m/r tablet |
| 1140861128 | tildiem 60mg m/r tablet |
| 1140860340 | timolol maleate+bendrofluazide 10mg/2.5mg tablet |
| 1140860342 | timolol maleate+bendrofluazide 20mg/5mg tablet |
| 1141194808 | timolol maleate+bendroflumethiazide 10mg/2.5mg tablet |
| 1140860336 | timolol maleate+co-amilozide 10mg/2.5mg/25mg tablet |
| 1140888496 | Torasemide |
| 1140864874 | torem 2.5mg tablet |
| 1140860172 | totamol 25mg tablet |
| 1140860904 | Trandolapril |
| 1141153328 | trandolapril + verapamil hydrochloride |
| 1140860334 | trasidrex tablet |
| 1140866328 | triam-co tablet |
| 1140866360 | triamaxco tablet |
| 1140866388 | Triamterene |
| 1140866324 | triamterene+benzthiazide 50mg/25mg capsule |
| 1141180772 | triamterene+chlortalidone 50mg/50mg tablet |
| 1140866330 | triamterene+chlorthalidone 50mg/50mg tablet |
| 1140866332 | triamterene+frusemide 50mg/40mg tablet |
| 1141195254 | triamterene+furosemide 50mg/40mg tablet |
| 1141188408 | tritace 1.25mg tablet |
| 1140926188 | unipine xl 30mg m/r tablet |
| 1140881692 | univer 120mg m/r capsule |
| 1140851336 | urizide 5mg tablet |
| 1141190548 | valni 20 retard 20mg m/r tablet |
| 1141201038 | valsartan+hydrochlorothiazide 80mg/12.5mg tablet |
| 1140851790 | vasad 5mg capsule |
| 1140866758 | vasaten 50mg tablet |
| 1140860892 | vascace 250micrograms tablet |
| 1141190160 | vascalpha 5mg m/r tablet |
| 1140851436 | vasetic co-amilozide 5/50mg tablet |
| 1141187774 | vera-til sr 120mg m/r tablet |
| 1140888510 | Verapamil |
| 1141150926 | verapress mr 240 m/r tablet |
| 1141169710 | vertab sr 240 m/r tablet |
| 1141151474 | viazem xl 120mg m/r capsule |
| 1140860338 | viskaldix tablet |
| 1140866108 | Xipamide |
| 1140866096 | xuret 500micrograms tablet |
| 1141153032 | zanidip 10mg tablet |
| 1141174684 | zemret 180 xl m/r capsule |
| 1141167832 | zemtard 120 xl m/r capsule |
| 1140860714 | zestril 2.5mg tablet |
| 1141171804 | zildil sr 60mg m/r capsule |
| 1141184390 | zolvera 40mg/5ml oral solution |

| **Supplementary Method Table 11. Codes used to identify participants with dyslipidaemia** | | |
| --- | --- | --- |
| UKBB code | **Code type** | **Code** |
| 1473 | UKBB SR | High cholesterol |
| NA | ICD9 | 272 Disorders of lipoid metabolism |
| NA | ICD9 | 272.0 Pure hypercholesterolaemia |
| 27200 | ICD9 | 272.00 Familial hypercholesterolaemia |
| 27201 | ICD9 | 272.01 Hyper-beta-lipoproteinaemia |
| 27202 | ICD9 | 272.02 Pure hypercholesterolaemia (hyperlipidaemia, group a) |
| 27203 | ICD9 | 272.03 Low-density-lipoid-type (ldl) hyperlipoproteinaemia |
| 27209 | ICD9 | 272.09 Pure hypercholesterolaemia (other) |
| 2721 | ICD9 | 272.1 Pure hyperglyceridaemia |
| 2722 | ICD9 | 272.2 Mixed hyperlipidaemia |
| 2723 | ICD9 | 272.3 Hyperchylomicronaemia |
| NA | ICD9 | 272.4 Other and unspecified hyperlipidaemia |
| 27240 | ICD9 | 272.40 Hyperlipidaemia of nephrotic syndrome |
| 27248 | ICD9 | 272.48 Hyperlipidaemia due to other specified causes |
| 27249 | ICD9 | 272.49 Hyperlipidaemia not otherwise specified |
| 2725 | ICD9 | 272.5 Lipoprotein deficiencies |
| 2726 | ICD9 | 272.6 Lipodystrophy |
| 2727 | ICD9 | 272.7 Lipidoses |
| NA | ICD9 | 272.8 Other disorders of lipoid metabolism |
| 27280 | ICD9 | 272.80 Other disorders of lipoid metabolism (lipase deficiency) |
| 27281 | ICD9 | 272.81 Other disorders of lipoid metabolism (steatosis) |
| 27282 | ICD9 | 272.82 Other disorders of lipoid metabolism (lipomatosis) |
| 2729 | ICD9 | 272.9 Unspecified disorders of lipoid metabolism |
| NA | ICD10 | E78 Disorders of lipoprotein metabolism and other lipidaemias |
| E780 | ICD10 | E78.0 Pure hypercholesterolaemia |
| E781 | ICD10 | E78.1 Pure hyperglyceridaemia |
| E782 | ICD10 | E78.2 Mixed hyperlipidaemia |
| E783 | ICD10 | E78.3 Hyperchylomicronaemia |
| E784 | ICD10 | E78.4 Other hyperlipidaemia |
| E785 | ICD10 | E78.5 Hyperlipidaemia, unspecified |
| E786 | ICD10 | E78.6 Lipoprotein deficiency |
| E788 | ICD10 | E78.8 Other disorders of lipoprotein metabolism |
| E789 | ICD10 | E78.9 Disorder of lipoprotein metabolism, unspecified |

| **Supplementary Method Table 12. Codes of lipid-lowering medications used to identify those with dyslipidaemia** | |
| --- | --- |
| Code | **Medication** |
| 1140861892 | Acipimox |
| 1141146234 | Atorvastatin |
| 1140861946 | atromid-s 500mg capsule |
| 1140861924 | Bezafibrate |
| 1141157260 | bezafibrate product |
| 1140861926 | bezalip 200mg tablet |
| 1140861928 | bezalip-mono 400mg m/r tablet |
| 1140862026 | Ciprofibrate |
| 1140861944 | Clofibrate |
| 1140861848 | colestid 5g/sachet granules |
| 1140888590 | Colestipol |
| 1140909780 | Colestyramine |
| 1141180734 | colestyramine product |
| 1141192414 | crestor 10mg tablet |
| 1140910632 | Eptastatin |
| 1141192736 | Ezetimibe |
| 1141192740 | ezetrol 10mg tablet |
| 1140861954 | Fenofibrate |
| 1141201306 | fibrazate xl 400mg m/r tablet |
| 1140888594 | Fluvastatin |
| 1140861856 | Gemfibrozil |
| 1141157262 | gemfibrozil product |
| 1140864592 | lescol 20mg capsule |
| 1141162544 | lipantil micro 67mg capsule |
| 1140861922 | lipid lowering drug |
| 1141146138 | lipitor 10mg tablet |
| 1140861970 | lipostat 10mg tablet |
| 1140861858 | lopid 300 capsule |
| 1141188546 | niaspan 500mg m/r tablet |
| 1140861868 | nicotinic acid product |
| 1140861894 | olbetam 250mg capsule |
| 1140888648 | Pravastatin |
| 1140861936 | questran 4g/sachet powder |
| 1141195196 | ranzolont 10mg tablet |
| 1141192410 | Rosuvastatin |
| 1141188146 | simvador 10mg tablet |
| 1140861958 | Simvastatin |
| 1141172214 | supralip 160mg m/r tablet |
| 1140910652 | Synvinolin |
| 1140910654 | Velastatin |
| 1140881748 | zocor 10mg tablet |
| 1141200040 | zocor heart-pro 10mg tablet |

**References:**

1. Alberti KG, Eckel RH, Grundy SM, et al. Harmonizing the metabolic syndrome: a joint interim statement of the International Diabetes Federation Task Force on Epidemiology and Prevention; National Heart, Lung, and Blood Institute; American Heart Association; World Heart Federation; International Atherosclerosis Society; and International Association for the Study of Obesity. *Circulation* 2009;120(16):1640-5.

2. Rinella ME, Lazarus JV, Ratziu V, et al. A multi-society Delphi consensus statement on new fatty liver disease nomenclature. *Hepatology* 2023.
